# Modular operation of microfluidic chips for highly parallelized cell culture and liquid dosing via a fluidic circuit board

**DOI:** 10.1038/s41378-020-00216-z

**Published:** 2020-11-30

**Authors:** A. R. Vollertsen, D. de Boer, S. Dekker, B. A. M. Wesselink, R. Haverkate, H. S. Rho, R. J. Boom, M. Skolimowski, M. Blom, R. Passier, A. van den Berg, A. D. van der Meer, M. Odijk

**Affiliations:** 1 BIOS Lab on Chip Group, MESA+ Institute for Nanotechnology, University of Twente, Enschede, The Netherlands; 2 Mesoscale Chemical Systems, MESA+ Institute for Nanotechnology, University of Twente, Enschede, The Netherlands; 3 Institute for Technology-Inspired Regenerative Medicine, Maastricht University, Maastricht, The Netherlands; 4 Micronit Microtechnologies, Enschede, The Netherlands; 5 Applied Stem Cell Technologies, TechMed Centre, University of Twente, Enschede, The Netherlands

**Keywords:** Engineering, Nanoscience and technology

## Abstract

Microfluidic systems enable automated and highly parallelized cell culture with low volumes and defined liquid dosing. To achieve this, systems typically integrate all functions into a single, monolithic device as a “one size fits all” solution. However, this approach limits the end users’ (re)design flexibility and complicates the addition of new functions to the system. To address this challenge, we propose and demonstrate a modular and standardized plug-and-play fluidic circuit board (FCB) for operating microfluidic building blocks (MFBBs), whereby both the FCB and the MFBBs contain integrated valves. A single FCB can parallelize up to three MFBBs of the same design or operate MFBBs with entirely different architectures. The operation of the MFBBs through the FCB is fully automated and does not incur the cost of an extra external footprint. We use this modular platform to control three microfluidic large-scale integration (mLSI) MFBBs, each of which features 64 microchambers suitable for cell culturing with high spatiotemporal control. We show as a proof of principle that we can culture human umbilical vein endothelial cells (HUVECs) for multiple days in the chambers of this MFBB. Moreover, we also use the same FCB to control an MFBB for liquid dosing with a high dynamic range. Our results demonstrate that MFBBs with different designs can be controlled and combined on a single FCB. Our novel modular approach to operating an automated microfluidic system for parallelized cell culture will enable greater experimental flexibility and facilitate the cooperation of different chips from different labs.

## Microfluidics: Circuit boards for flexible, modular design

Researchers in the Netherlands have developed a fluidic circuit board (FCB) platform which can be used to operate several microfluidic building blocks (MFBB). Highly integrated microfluidic chips are challenging to design and are customized for specific applications, limiting the possibility of re-use. A team led by Mathieu Odijk of the University of Twente sought to make flexible design alterations of microfluidic circuits possible. To that end, they developed a modular FCB platform incorporating an MFBB enabler. The enabler can operate the MFBBs in parallel or selectively and can also “save” the state of an MFBB. As a proof-of-concept, the team used their platform to combine a dosing unit and a module with 64 parallelized culture chambers. These new tools offer researchers the flexibility to combine different microfluidic chips and easily create parallelized versatile culturing systems.

## Introduction

Massively parallelizing microfluidic cell culturing is essential for expanding the parameter screening space and increasing throughput in a wide variety of biological applications. These applications include drug screening^1–4^, cell transfection^5^, cell signaling pathway mapping^6^, stem cell differentiation^7–9^, and stem cell generation^10^. Common approaches for screening a large parameter space are droplet microfluidics^11,12^, microfluidic gradient generators^2,13^, and microfluidic large-scale integration (mLSI) chips^7,9,14^. However, only the last of these three methods is also suitable for parameter screening in a dynamic, temporally controlled manner. Temporal control is crucial for maintaining tight control over the cell microenvironment^15^, and is, therefore, an essential factor to take into account when designing massively parallelized microfluidic cell culture systems.

mLSI chips contain hundreds to thousands of integrated microvalves and were first developed by Thorsen et al. as the microfluidic counterpart of the integrated circuit^14^. Previously, similar to electrical engineers in the 1950s and 1960s, microfluidic engineers were confronted with the practical limitations of creating chips with a higher level of integration. For example, increasing throughput was only possible by increasing the number of replicates, which in turn increased the amount of external equipment (e.g., pumps) and tubing connections required. This problem was referred to as the “tyranny of numbers”^16^. The invention of the microvalve (analogous to the transistor)^17^ by the Quake group and its integration into mLSI chips largely solved this problem. By using microvalves to create on-chip multiplexers, Sjoberg-Gomez et al. and Wu et al. have demonstrated mLSI chips with 96 and 128 independently addressable cell culture chambers, respectively^7,9^. Recently, Zhang et al. demonstrated a chip with an impressive number of 1500 independently addressable chambers^18^. However, such highly integrated chips are challenging to develop and set up, as this requires a complex design cycle, custom software for chip-specific operation and a highly optimized operating protocol. As a consequence, flexible alterations to the design of these monolithic chips are not easily realizable when required by the experimental question. To address this challenge in maintaining design flexibility while setting up a highly parallel mLSI cell culture system, we propose a modular approach to create a versatile system based on a library of standardized components.

We have previously reported a modular platform for microfluidics in which a single fluidic circuit board (FCB) connects multiple microfluidic building blocks (MFBBs) in a modular and standardized fashion^19,20^. Analogous to the printed circuit board, the predefined MFBBs can be mounted onto the FCB and connected through the FCB to fit a customized purpose. Other modular microfluidic systems^21–24^ have been previously reported, but these systems rely on directly connecting MFBBs to each other or on integrating all MFBBs in a microfluidic breadboard and selecting them by mounting passive chips^25^. In contrast, our FCB provides a single base plate through which multiple MFBBs can be controlled and connected in a modular fashion^19,20^. Importantly, the MFBB and FCB format and interface, standardized by the ISO WA (workshop agreement)^26^, provide a framework within which further MFBBs can be designed to fit the FCB. In this way, MFBB designs with established protocols can be combined and upscaled. Moreover, FCB fabrication can be outsourced so that only custom MFBBs with unique functions are made in-house.

Here, we extend our FCB MFBB technology by developing the first FCB that contains an active function: an MFBB enabler. We use this FCB to operate mLSI MFBBs both in parallel and selectively and hereby present, to the best of our knowledge, the first modular plug-and-play system for mLSI chips. Furthermore, we demonstrate the versatility of the FCB by using the same FCB to control two MFBBs, an mLSI MFBB and a dosing MFBB, which have entirely different architectures. Importantly, we show that the FCB MFBB enabler can “save” the states of the valves in the mLSI MFBB. This feature allows us to combine both the dosing and the mLSI MFBB into a single system while operating both MFBBs using a shared set of control lines via the FCB. Furthermore, we show as a proof of principle that we can culture human umbilical vein endothelial cells (HUVECs) in the chambers of an unmounted mLSI MFBB as a first step toward applying this modular technology to create automated and highly parallelized yet versatile cell culture systems.

## Design

The dimensions of both the mLSI and the dosing MFBBs, as well as of the FCB, are in accordance with the standards defined in ISO WA 23:2013^26^. In addition, all inlets on the FCB, and consequently all MFBB control channel inlets, are located as points on a 1.5-mm grid also defined in ISO WA 23:2016^26^. By adhering to these standards, potential system extension to include other likewise standardized components is facilitated.

### 64-Chamber mLSI MFBB for parallelized cell culture

**Fig. 1:**
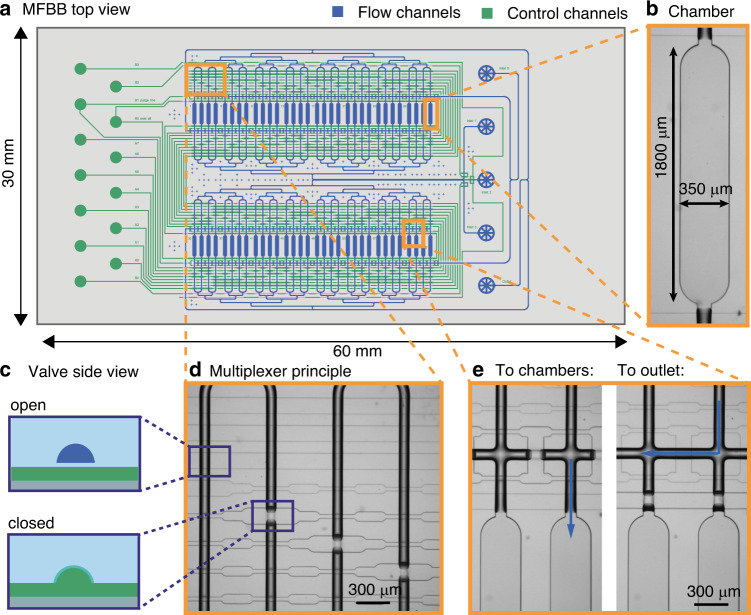
Design and operation of the mLSI MFBB. **a** Schematic design of the mLSI MFBB. The channels in the flow layer are shown in blue, while the channels in the control layer are shown in green. **b** Brightfield micrograph of a chamber. **c** Schematic side view of a valve in normally open “push-up” configuration. **d** Brightfield micrograph illustrating the multiplexing principle for the first four chambers. **e** The brightfield micrograph on the left shows the bypass channel blocked by valves, whereby the flow is directed into the chambers. The right micrograph shows the chambers blocked by valves, and the bypass channel opened. As a result, the flow is directed to the outlet, purging the channels without mixing with the chamber content

This mLSI MFBB is designed to screen up to 64 different conditions in a spatiotemporally independent manner. It contains 64 independently addressable chambers, a bypass channel, two outlets and three independently addressable inlets, as shown in Fig. 1a. The chambers and all flow channels are shown in blue, while the control channels are green. Fig. 1b represents a brightfield micrograph of one of the chambers. Flow from the inlets is directed within the chip by selectively opening and closing normally open valves^14^ in the “push-up” configuration (Fig. 1c). Since the control channels are dead-ended, pressurizing these channels causes the flexible membrane to deflect into the flow channel, effectively blocking the flow. Flow channels in places where there are valves have a rounded profile so that they can be closed without leakage. This profile makes the (air-filled) channels appear darker than channels with a rectangular cross-section in brightfield micrographs. The control channels are filled with water to prevent air from permeating the valve membrane and consequently forming bubbles in liquid-filled flow channels. All chambers can be independently addressed using a combinatorial multiplexer^27^. The number of independently addressable chambers *k* depends on the number of control channels *N* as follows:

1 $$k= \frac{N!}{(N/2)!^2}\; \; \; \; \; \; \; for \, N(\in 2{\mathbb{N}})\, {and}$$

2 $$k= \frac{N!}{((N+1)/2)!((N-1)/2)!}\;\;\;\;\;\;\; for \, N(\in 2{\mathbb{N}}_0 + 1)$$

In this case, *N* = 8 control channels are used, with which, in theory, 70 chambers can be independently addressed. However, we opted for 2^6^ = 64 chambers because it allows the connecting channels to be binarily branched from the in- and outlet (the branched flow channel structure is visible in the top and bottom of the design in Fig. 1a). This results in equal flow resistance for all chambers regardless of whether they are all opened simultaneously or individually. The multiplexing principle is visualized in Fig. 1d, which shows four flow channels (dark appearance in image) crossed by eight control channels (light appearance). The pressure in the top four control channels is atmospheric; therefore, the valves are open. The bottom four control channels are pressurized, thereby closing the valves. Due to the arrangement of the valves (wide cross-sections) and bridges (narrow cross-sections), only the first flow channel is unobstructed. Pressurization of any four of the eight control channels results in exactly one chamber being accessible while the paths to all others are obstructed (disregarding the six leftover combinations which are not used in this case). The bypass channel is used to purge the channels without contaminating the chambers when switching liquids. Therefore, it is located just outside the chambers where it runs across all of the branched flow channels. The valves of two more control channels are used to direct the flow either into the chambers or through the bypass channel (Fig. 1e). Finally, three valves are used to open or close three flow channel inlets (as shown at the channel junction, where the three inlet channels meet in Fig. 1a). In total, each mLSI MFBB has 13 control channels controlling a total of over 700 valves. Further details on the working principle of this MFBB are described in Fig. S1.

The described design is realized in a three-layer MFBB 3 cm × 6 cm in size. The top two layers are the flow layer (blue in Fig. 1a) and the control layer (green in Fig. 1a), whereas the third layer seals the control layer and interfaces the MFBB with the FCB. Two variations of the design described above were fabricated, with minor differences. These differences are shown and explained in detail in Fig. S2. The reason for the minor adjustments was to improve the MFBB for cell culture.

### Liquid dosing MFBB with a high dynamic range

Various operational principals can be used to accomplish defined microfluidic liquid dosing, including peristaltic pumping^28^, electrowetting on a dielectric^29^, fixed-volume reservoir metering^30^, and pulse width modulation (PWM) metering^31,32^. This MFBB applies the concept of PWM to accomplish microfluidic dosing so that concentration profiles with a wide range of mixing ratios (i.e., high dynamic range over 1–2 orders of magnitude) within a short time period (tens of seconds) can be generated.

**Fig. 2:**
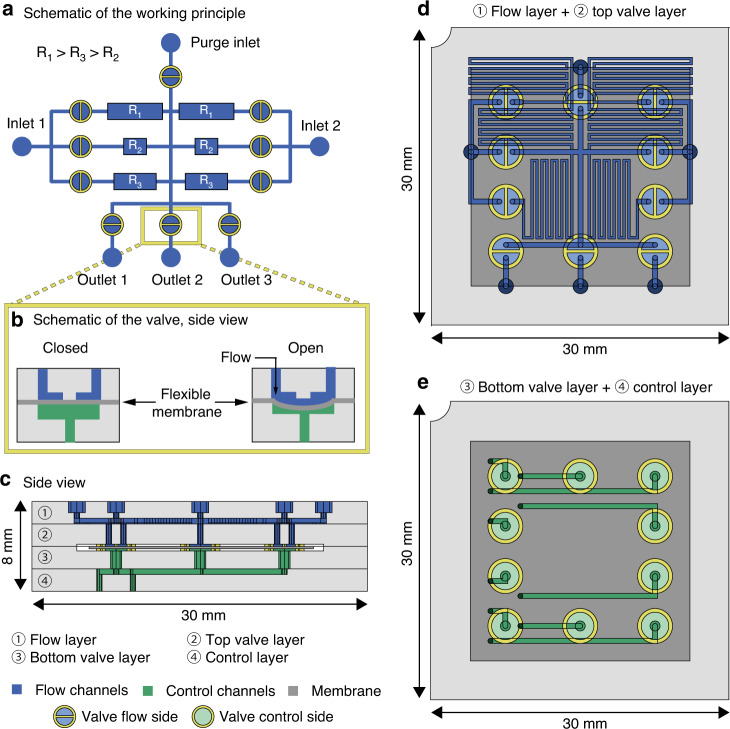
Design and operation of the dosing MFBB. **a** Schematic diagram of the MFBB working principle. **b** Schematic side view of the integrated valves. **c** Realistic side view of the MFBB showing all four layers. **d** Realistic top view of the flow layer, containing the in- and outlets, the hydraulic resistors (layer ①) and the top part of the valves (layer ②). **e** Realistic top view of the control layer, containing the control channels (layer ③) and the bottom part of the valves (layer ④)

A schematic overview of the working principle is shown in Fig. 2a. The MFBB contains two fluid inlets, one purge inlet, three fluid outlets, and ten integrated valves in a normally closed configuration that can be actuated via the control lines (shown schematically in Fig. 2b). The valve design is a modified version of a design by Loessberg-Zahl^33^. Each of the fluid inlets is connected in parallel to three channels with different hydraulic resistances. The fluid can be routed through any of these three channels using the valves. As a result, three different flow rates per inlet and applied pressure can be obtained, as described in the following equation:

3 $${Q_n}=\Delta {PR_{n}^{-1}} \quad n \in \{1,2,3\}$$

where *Q* (m^3^ s^−1^) is the flow rate, ∆*P* (Pa) is the pressure over the channels, and *R* (Pa s m^−3^) is the hydraulic resistance. The large differences between *R*_1_, *R*_2_, and *R*_3_ allow for a high dynamic range in the dosed volume per time unit. By extension, a high dynamic range in concentration is achieved when the dosed volume from inlet 1 is combined with fluid from inlet 2 (or vice versa). In theory, different concentration profiles can be generated by combining flow from the two fluids that are routed through two different hydraulic resistors. However, in this steady-state approach, the number of concentration profiles is limited by the number of resistor combinations. Therefore, the valves are opened with defined pulse widths to modulate the fluid volume from each inlet, thereby generating many different concentration profiles. After microfluidic dosing, the mixture is then directed to one of the outlets and homogenized by Taylor dispersion in the connected tubing. One dosing MFBB can output fluid mixtures to up to three subsequent MFBBs (one per outlet), which then receive the fluid mixture as an input. Finally, the purge inlet serves to clear the channels (and optionally the tubing) with a neutral fluid.

The described design is realized in a four-layer MFBB 3 cm × 3 cm × 0.8 cm in size (Fig. 2c–e). The top two layers (① and ② in Fig. 2c, d) contain the flow channels (blue), which are connected to the in- and outlets. The bottom layers (③ and ④ in Fig. 2c, e) contain the control channels (green), which control the valve actuation. The top and bottom parts of the valves (in layers ② and ③, respectively) are separated by a flexible membrane (dark gray in Fig. 2b, d, e), which is situated between layers ② and ③.

### Fluidic circuit board

**Fig. 3:**
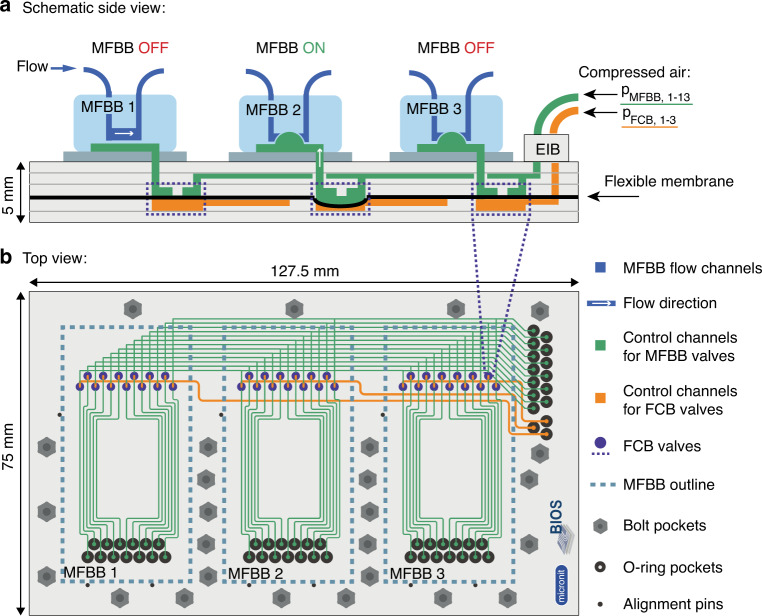
Design and operation of the FCB and connected MFBBs. **a** Schematic side view of the FCB and three MFBBs. The MFBB control channels (green) are operated via the FCB. The FCB control channels (orange) can block pressure transmission to an MFBB. If the FCB valve of an MFBB is closed (open), the MFBB is disabled (enabled) or OFF (ON). **b** Top view of the FCB. Each of the 13 MFBB control channels branches off from a common inlet to 3 MFBB ports. Each set of channels is controlled by a set of FCB valves (purple)

The purpose of this FCB is both the parallelized and sequential operation of MFBBs, specifically their control layers. Figure 3a shows a schematic of the operating principle in a side view. The MFBB control tubing (green), which would usually be directly attached to a single MFBB, is connected to the FCB via a plug-and-play external interconnection block (EIB). The schematic shows one MFBB control channel (green) that is branched in the FCB into three channels, each of which leads to a different MFBB. In this way, three identical MFBBs can be operated in parallel. For independent MFBB operation, the concept of latching valves is applied^34^. Specifically, there is one set of valves per MFBB that can close off all of the control channels of the MFBB. Each set of valves is controlled by one FCB control channel (orange). If a set of FCB valves are closed, pressure changes in the MFBB control channels cannot be transferred to the corresponding MFBB. This MFBB is then effectively disabled (but retains its last valve states), and another MFBB can be operated independently of the first. To illustrate the working principle, Fig. 3a shows three different states of MFBB operation. MFBB 1 is disabled (OFF) with the MFBB control channel depressurized. As a result, flow can pass through MFBB 1, but its control valves can no longer be operated through the FCB. MFBB 2 is enabled (ON), meaning that the MFBB valves can be operated through the FCB. Here, the MFBB valve is closed since the MFBB control channel is pressurized. Finally, MFBB 3 is disabled (OFF), similar to MFBB 1. However, in contrast to MFBB 1, MFBB 3 was disabled when the MFBB control channel was pressurized. Therefore, the valves in MFBB 3 are closed, and there is no flow through the corresponding flow channel. Since the three FCB control channels are fully independent of each other, it is possible to select any one, two, or all three MFBBs to be enabled simultaneously.

Figure 3b shows an accurate top view of the FCB. The FCB footprint fits that of a microtiter plate, which makes it compatible with commercially available microscope stage inserts. The FCB has ports for three MFBBs with a maximal footprint of 3 cm × 6 cm each. There are pockets for three alignment pins per MFBB for easy alignment of the FCB-MFBB control channel interface. Since O-rings are used for a leak-tight seal, the FCB has O-ring pockets that allow for a compression of 10% and keep the MFBB level on the FCB. The EIB is fastened to the FCB directly using bolts and nuts. The MFBBs are fastened likewise using clamps. A detailed design of the EIB and clamps is shown in Fig. S3. The FCB has hexagonal pockets on the bottom side to inset the bolt heads. The bolts are inserted through the FCB and EIB or clamp and then tightened with nuts at the top. In the top view, the complete set of FCB valves (purple) is also shown. There are 13 valves per set, as there are 13 MFBB control channels. The branching off of the main MFBB control channels is realized by a bridging layer in the FCB. In total, the FCB has five layers, excluding the flexible membrane (Fig. 3a).

## Results and discussion

### Fabrication results

**Fig. 4:**
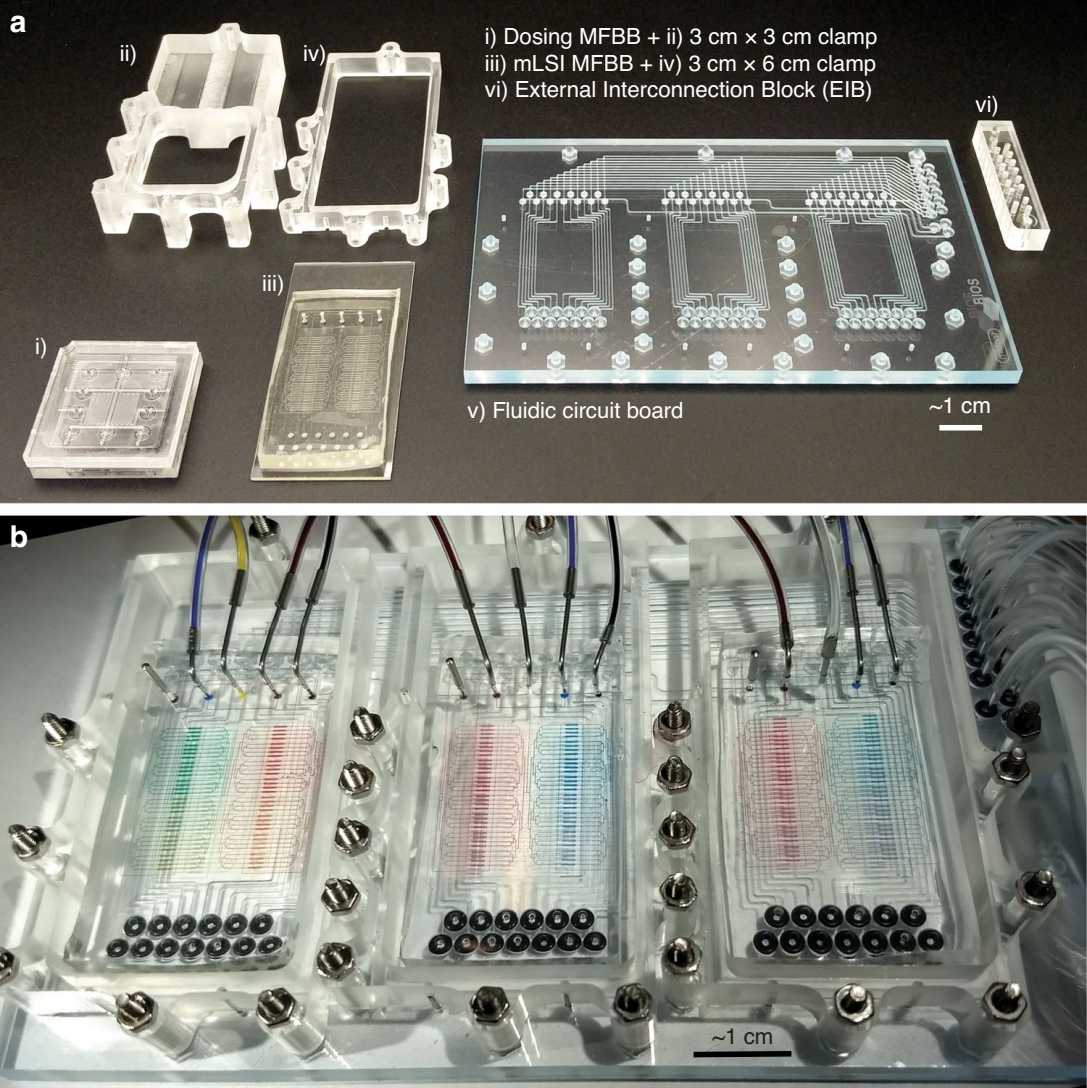
Components of the modular platform. **a** Fabricated parts for the platform assembly, numbered as follows: (i) Dosing MFBB, (ii) clamp (3 cm × 3 cm) for the dosing MFBB, (iii) mLSI MFBB with 64 chambers, (iv) clamp (3 cm × 6 cm) for the mLSI MFBB, (v) fluidic circuit board, and (vi) EIB. **b** Fully assembled platform with three mLSI MFBBs filled with food coloring gradients

An mLSI MFBB, a dosing MFBB, their clamps, the FCB, and the EIB are shown separately in Fig. 4a. The mLSI MFBB is fabricated from two polydimethylsiloxane (PDMS) layers bonded to a glass slide that has through-holes for the control channel inlets. The dosing MFBB is fabricated from four layers of poly(methyl methacrylate) (PMMA). The FCB consists of five polystyrene layers and a styrene ethylene butylene styrene (SEBS) membrane. The clamp and the EIB are fabricated from PMMA. Figure 4b shows the fully assembled platform with three mLSI MFBBs. The chambers are filled with food coloring to visualize the chambers and channels. Tubing for both the FCB and the MFBB control channels can be seen attached to the EIB, while tubing for the MFBB flow channels is connected to the MFBBs directly. In this way, each mLSI MFBB can still be filled with different solutions (cell suspensions, media, etc.), thus maintaining the versatility of having three separate mLSI MFBBs. The FCB, clamps and EIB are reusable, whereas the MFBBs that are used for cell culturing are created for single use. Finally, the FCB was fabricated in an industrial setting (at Micronit Microtechnologies), while the MFBBs, clamps and EIB were fabricated in an academic lab. The standardized interfaces and formats greatly facilitate collaboration between industry and academia, enabling a faster path to commercialization of the reusable components.

The assembly of the MFBBs on the FCB is simple and depends on screwing nuts and bolts into the clamps that hold the MFBBs in place, thus creating leak-proof interfaces between the inlets of the MFBBs and the outlets of the FCB via O-rings. However, in our FCB, the O-ring pockets are marginally too deep to reliably give sufficient O-ring compression at all interconnects every time an MFBB is mounted. Due to the minimal compression and close proximity of the O-rings, even slight variations in O-ring thickness can lead to a thinner O-ring not sealing sufficiently when it is placed next to thicker O-rings. As a result, the water in the MFBB control channel leaks out at the O-ring, and the valves in the MFBB do not close fully. Nevertheless, we succeeded in mounting three mLSI MFBBs leak-free by rearranging the O-rings based on their thickness and carefully tightening the clamp. We expect that the reliability of O-ring seals can be greatly improved in the next FCB generation by designing for at least 15% compression instead of 10%, which will also shorten the time required for platform assembly.

### Mono-type MFBB operation via the FCB

Three mLSI MFBBs were mounted onto the FCB. For the following FCB operation, the MFBB control channels were switched between 1.4 bar for closed and 0 bar for open MFBB valves. The FCB control channels were switched between 1.6 bar for closed FCB valves and −200 mbar for open FCB valves. The closing behavior of one set of 13 FCB valves is shown in Fig. S4 at a pump pressure of 1.4 bar and different gate pressures.

**Fig. 5:**
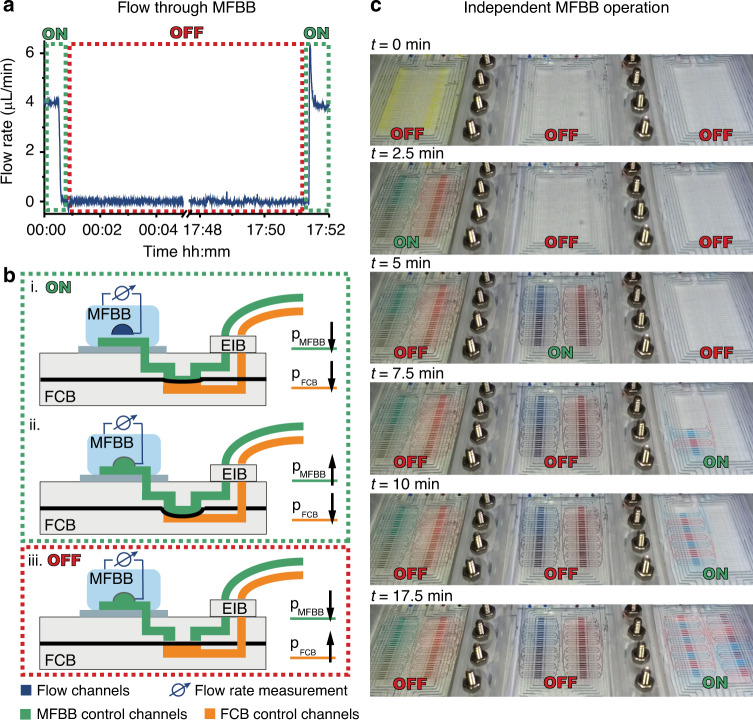
Operation of MFBBs through the FCB by sequentially disabling the MFBBs while ‘saving’ the states of their control channels. **a** A flow channel in an MFBB was opened, and the flow through the channel was measured. After 1 min, the flow was stopped by pressurizing the control channel in the MFBB through the FCB. Then, the MFBB was disabled, and the pressure to the FCB for the MFBB control channels was released. After more than 17 h, the MFBB was re-enabled, and the flow channel was reopened. **b** Schematic representation of enabling (ON) and disabling (OFF) the MFBB. **c** Video frames of sequential MFBB operation

The FCB is designed to “save” the current states of the MFBB valves after an MFBB is disabled. However, due to the gas permeability of the mLSI MFBB, water from pressurized control channels can ultimately leak out of the channels in the form of water vapor^35^. As a result, the pressure in the control channels decreases, and the valve membrane sealing off the flow channel slowly relaxes back to its native state. Once the membrane has relaxed enough for the valve to become leaky, the MFBB has to be enabled and the control channel repressurized. Therefore, the MFBB usage time depends on how long the pressure in the control channels of a disabled MFBB can be retained. If the pressure in the control channels decreases too quickly, processes in another MFBB would have to be interrupted to repressurize the control channels of the first MFBB. Therefore, we measured the flow of deionized (DI) water through an MFBB mounted on the FCB (Fig. 5a). First, the MFBB was enabled with open valves (a schematic of the principle is shown in Fig. 5b (i). Subsequently, the valves were closed (Fig. 5b (ii)) the MFBB was disabled, and then the MFBB control channel pressure was released (Fig. 5b (iii)). As shown by the flow rate in Fig. 5a, the MFBB valves remained closed for the entire duration of the experiment (>17 h). When the MFBB was re-enabled and the valves opened, the flow rate returned to the initial flow rate of 4 µL/min. The peak shown in Fig. 5a upon depressurization of the control channels is attributed to the pull created by all of the valves in the MFBB opening simultaneously, which can be circumvented by depressurizing the control channels one by one. Furthermore, the flow rate was measured through channels that were set to remain open when the MFBB was disabled. After 16 min, the flow rate was still at 95% of the initial flow rate (shown in Fig. S4).

Individual MFBB operation is demonstrated in Fig. 5c, where the MFBBs are sequentially filled with food coloring. The first two MFBBs are filled with all three inlets open simultaneously. The three colors are partially mixed by diffusion as they reach the channel branches. As a result, the chambers in these MFBBs are filled with a food coloring gradient. The third MFBB is filled chamber by chamber, alternating and mixing the blue and red food coloring. A full video of the operation on the FCB is shown in Video S1.

Although the total number of independently addressable chambers is not as high as in monolithic systems previously presented in the literature, the modular approach gives the user more freedom in tailoring the system to experimental requirements. For example, if the aim is to perform preliminary testing to identify a promising concentration range for the desired efficacy of a new compound, a system with hundreds of chambers is unnecessary. In the modular approach, the user can choose between using only one, two, or all three of the mLSI MFBBs to suit their aim, without having to design, fabricate and program a new device. In an analogy to standard cell culture in microtiter plates, this system can be considered as offering the ability to switch between microtiter plates with, e.g., 6-, 48-, or 96-wells.

### Multi-type MFBB operation via the FCB

**Fig. 6:**
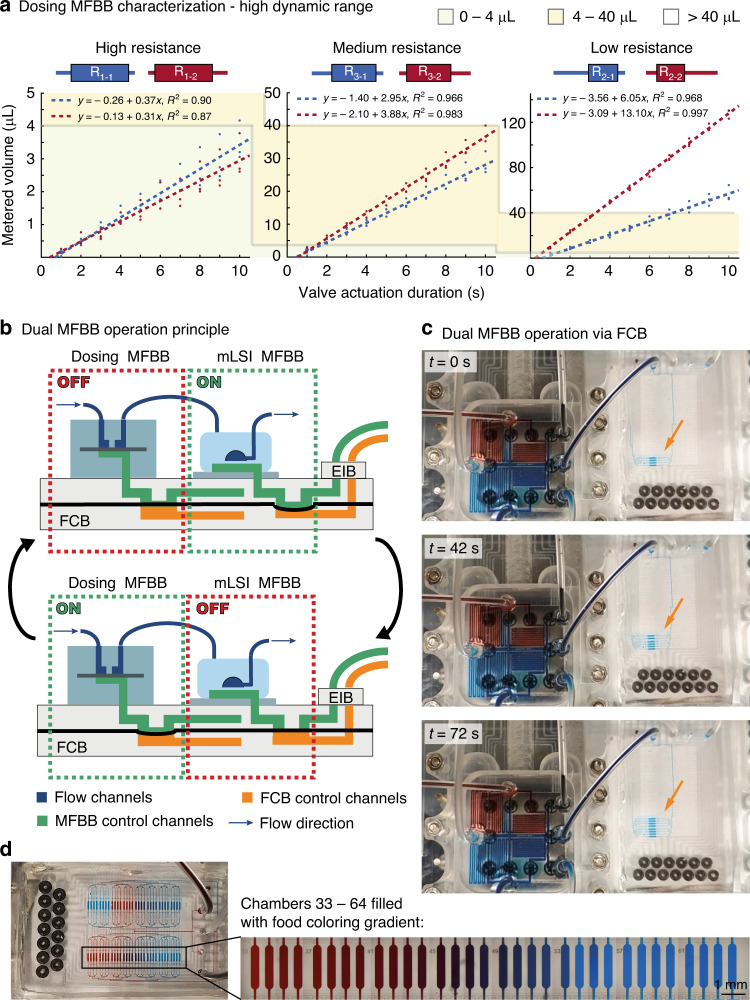
Operation of two different MFBBs (a dosing and an mLSI MFBB) via the FCB. **a** Dynamic range characterization of the dosing MFBB in terms of flow rate through the three hydraulic resistors at a pump pressure of 300 mbar. **b** Schematic of the dosing MFBB and the mLSI MFBB operated via the FCB. By sequentially enabling the MFBBs, the dosing MFBB can be used to fill the chambers of the mLSI MFBB. **c** Video frames showing the two MFBBs on the FCB and connected to each other with tubing. **d** Chambers of the mLSI MFBB filled with red or blue food coloring (which were selected in the dosing MFBB) or gradients generated by a long pulse of one food coloring followed by a long pulse of the other food coloring (chambers 33–64). The close-up view of chambers 33–64 consists of stitched brightfield micrographs

To demonstrate that two MFBBs with very different architectures (regarding both design and materials) can be operated by the same FCB, a dosing and an mLSI MFBB were mounted onto the FCB. In the first step, the dosing MFBB was characterized by measuring the metered volume of DI water with a pump pressure of 300 mbar through the high, medium and low hydraulic resistance channels for a duration of 1–10 s (Fig. 6a). The dynamic range of the system is demonstrated by the three different volumetric metering regimes (0–4, 4–40, and 40–140 µL) in which the MFBB can operate. The plots show linear behavior, which allows for a predictable volume to be metered through a combination of pulses from the high-, medium-, and low-resistance channels. The MFBB is capable of metering volumes less than 1 μL using the high-resistance channel and up to 128 μL in 10 s using the low-resistance channel in array 2 (red). In a 30-s period, this full range can be covered, yielding a dynamic range of at least 1:128. This value was lower for resistor array 1 (blue) due to bubbles trapped in the low-resistance valve, and as such, a dynamic range of at least 1:56 was achieved. If more time is allowed for profile generation, then a higher dynamic range can be achieved. An extensive characterization of this MFBB at different pump pressures and experimentally determined hydraulic resistances is presented in Figs. S5–S8 and Table S1.

In the next step, the MFBBs were combined by connecting one of the outlets of the dosing MFBB to an inlet of the mLSI MFBB with tubing. A flow pressure of 270 mbar was applied to the food coloring at the inlets of the dosing MFBB, and a pressure of 2.0 bar was used to operate both the MFBB and FCB valves. Dual operation of both MFBBs on the same FCB was achieved by successively alternating which MFBB was enabled (schematically shown in Fig. 6b). When the mLSI MFBB was enabled, a designated chamber was opened, and the MFBB valve states were saved by disabling the MFBB. Then, the dosing MFBB was enabled, and red or blue food coloring was selected by opening the corresponding valves. The food coloring then flowed into the mLSI MFBB, filling the open chamber. Figure 6c shows video frames (Video S2) of chambers 5–8 being filled in this manner. Figure 6d shows the mLSI MFBB with all chambers filled. Chambers 33–61 are filled with a food coloring gradient generated by a long red pulse following a long blue pulse (the chambers were filled in reverse order). Fully purging the tubing between the two MFBBs of its content (approximately 8 µL) took approximately 7.5 min. The comparatively large dead volume of the tubing is a drawback of the current FCB. In future FCB generations, we will connect the MFBBs via channels in the FCB, which will allow us to reduce the dead volume to approximately 1.5 µL (80% reduction). This connection will decrease the total filling time and save reagents.

### Cell culture in the chambers of the mLSI MFBB

The mLSI MFBB is designed to be suitable for multiplexed cell culture. As a proof of principle, HUVECs were cultured in the chambers of an unmounted mLSI MFBB. In this case, the glass slide of the MFBB did not have through-holes; instead, the control channel inlets were punched through the PDMS from the top. The MFBB was prepared for cell seeding by coating the flow channels with PLL-PEG (100 µg/mL in phosphate-buffered saline (PBS)) to reduce cell adhesion and coating the chambers with collagen I (0.1 mg/mL in PBS) to promote cell adhesion. After the cells were seeded in the chambers, a program was set to exchange the cell medium chamber by chamber every 3 h.

**Fig. 7:**
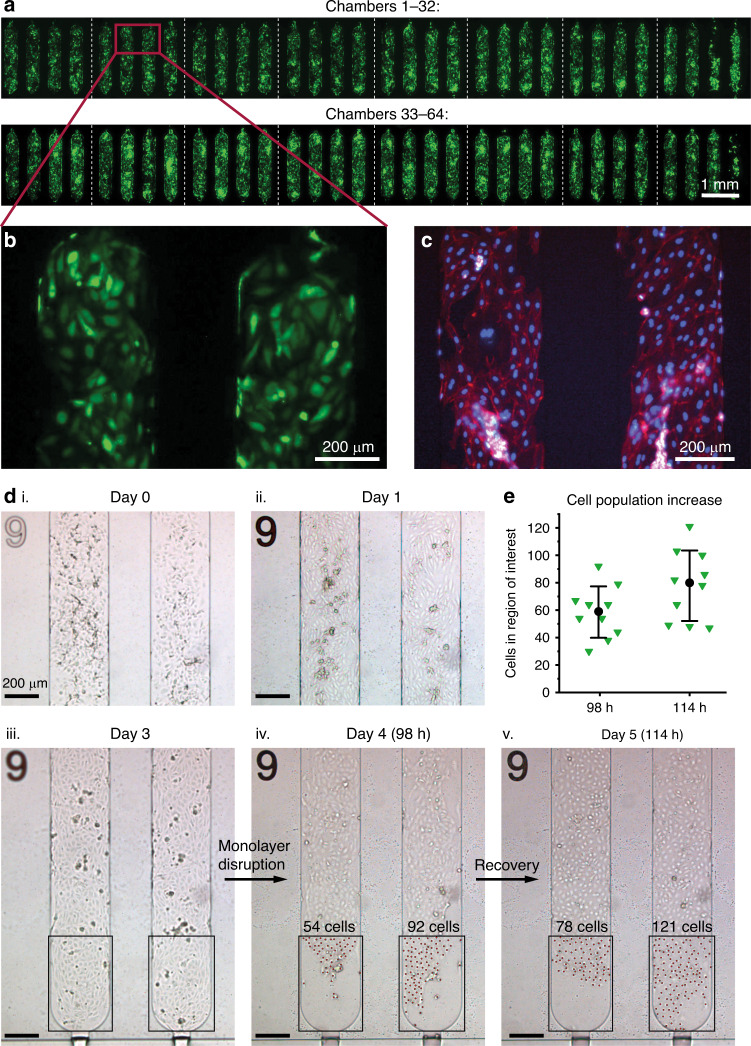
Proof-of-principle HUVEC culture in an unmounted mLSI MFBB. **a** Live-cell fluorescence images (one image per four chambers) showing an overview of all the chambers after 3 days of culturing GFP-expressing HUVECs. **b** Live-cell fluorescence image of GFP-expressing HUVECs in chambers 6 and 7. **c** Fluorescence image of fixed HUVECs with the cell F-actin and nuclei stained with ActinRed and NucBlue, respectively. **d** HUVECs after seeding and subsequent monolayer formation. The cells were seeded at a high cell density (i) and confluent on day 1 (ii). The monolayer was still intact on day 3 (iii) but began to deteriorate on day 4 (iv). At this point, the medium in the supply vial was replaced. The cells showed signs of recovery on day 5 (v) as the monolayer started to reform. The red dots in (iv) and (v) mark the cells counted in the region of interest. **e** Cells in ten chambers were counted in regions where the monolayer had deteriorated on day 4 (98 h) and after 16 h of recovery (114 h). The black dots represent the mean cell count, and the error bars represent the standard deviation. The cell number increased by an average of 33% in these areas

Figure 7a shows an overview of all chambers after 3 days of cell culture. The figure consists of 16 live-cell fluorescence images (one per four chambers) where the HUVECs are visualized by green fluorescent protein (GFP) expression. The figure confirms that there are HUVECs present in all chambers. Figure 7b shows the HUVECs in chambers 6 and 7 at a higher magnification. Figure 7c shows HUVECs from a different experiment (non-GFP-expressing cells in this case), where cells were fixed after 2 days of culture. The F-actin filaments and nuclei of the cells were visualized by ActinRed and NucBlue staining, respectively. The results show that the cells had grown together to form the cobblestone-like morphology typical for HUVECs.

Figure 7d shows brightfield images of HUVECs cultured in two chambers (9 and 10) over the course of 5 days. The cells were seeded with a high cell number per chamber (Fig. 7d (i)) such that confluency was reached overnight (day 1, Fig. 7d (ii)). On day 1, the cells were more elongated than immediately after seeding and had started to fill in any empty gaps that were left after seeding. By day 3 (Fig. 7d (iii)), the cells had formed a confluent monolayer in the chambers. This monolayer is typical for healthy HUVECs. However, at the end of day 4 (Fig. 7d (iv)), the monolayer became disrupted with the formation of large gaps. At the bottom of the chambers, significant cell death was visible, accompanied by a receding monolayer. It was hypothesized that the cell stress was due to degradation of the medium in the vial connected to the MFBB inlet. Therefore, the vial was replaced with one containing fresh medium. On day 5 (16 h later), the cells showed signs of recovery as they reformed the monolayer.

To give an indication of how quickly the monolayer recovers at its edges, the cells at the monolayer edges were manually counted on days 4 and 5. Areas of the same size were chosen in ten different chambers, e.g., as shown in Fig. 7d (iv) and (v) by the black rectangle around the region of interest. Each area contained a few dozen cells on day 4, but these covered at most half of the area. The cell counts are shown in Fig. 7e, whereby the black dots and error bars represent the means and standard deviations, respectively. On average, the cell population in these areas increased by 33 ± 17% within 16 h.

## Conclusion and outlook

The described platform consisting of the FCB and MFBBs is, to the best of our knowledge, the first modular plug-and-play system for mLSI chips. By integrating an MFBB enabler into the FCB, we can operate up to three of the same MFBBs in parallel or operate and combine different MFBBs with different operation protocols. The standardized interface with clamps and O-ring connections allows for different MFBBs fabricated by different methods to be combined in a single system, as demonstrated with our micro-milled dosing MFBB and soft lithography-based mLSI MFBB. Our modular approach toward creating automated, highly parallelized cell culturing systems will give the end-users more flexibility in several aspects. First, this system provides flexibility in redesigning the microfluidic chips since only the layers in the MFBB are affected. As long as the new MFBB retains the same interface and a standardized format, it can be operated via the same reusable FCB. Second, with this system, it becomes possible to run different experiments on different chips (e.g., with design criteria tailored to different cell types or cell constructs) simultaneously without having to use multiple pneumatic control setups for the mLSI chips. Furthermore, it is possible to exchange MFBBs on the FCB to adjust the system to a new application.

In this article, we demonstrated the technical functionality of our system. Currently, we are working on the development of further MFBBs and on using the mLSI MFBB for stem cell differentiation. In addition, we plan to improve our platform by making it more broadly compatible with automated imaging systems. At present, special microscope objectives with long working distances are needed to image through the FCB. We plan to solve this problem by removing parts of the FCB underneath the MFBB regions of interest and by reducing the overall FCB layer thickness.

In the future, modular MFBBs may also be conceptually combined with previously reported FCBs^19,20^, which can route samples from one MFBB to another, decreasing dead volume. Such a modular platform will greatly facilitate cell culture applications in which MFBBs with different functions (e.g., mixers, sensors, different cells, or tissues) are connected and integrated. In the future, we expect that this flexible integration of MFBBs will also be particularly useful to control and integrate advanced microfluidic cell culture models known as organs-on-chips^36,37^, as well as their linking into multiorgan “body-on-a-chip” systems^38^. Overall, our technology provides a powerful yet versatile toolset for microfluidic cell culture applications.

## Materials and methods

### 64-Chamber mLSI MFBB

The mLSI MFBB was designed in CleWin Layout Editor (version 4.3.6.0). The 64 chambers each measured 1.85 mm × 0.35 mm in length by width and had rounded corners. The design for the flow layer was scaled by a factor of 1.01 to compensate for PDMS shrinkage. Furthermore, the valve and bridge designs included tolerances of a few tens of micrometers to facilitate later alignment of the flow and control layers.

For each mLSI MFBB type (see Fig. S2), two wafer molds, one for the control layer and one for the flow layer, were prepared by standard photolithography. For the control layer, a wafer coated with SU8 (MicroChem, USA) was used to create 20-µm-high channels. For the type II flow layer, a wafer coated with SU8 (MicroChem, USA) was first used to create rectangular channels approximately 48 µm high in the places where there are no valves in the design. Next, AZ40XT (MicroChemicals, Germany) was used to create channels with a rounded profile, approximately 35 µm high. Channel heights were measured with a Dektak^®^ stylus profiler (Veeco, Germany). For the type I flow layer wafer, all structures were created using AZ40XT photoresist.

Both types of mLSI MFBBs were fabricated by multilayer soft lithography^17^. A PDMS (RTV615, Permacol, The Netherlands) offset ratio was used to bond the flow (1:7 w/w, curing agent to base polymer) and control (1:20 w/w, curing agent to base polymer) layers together. The PDMS was mixed, degassed and poured over the respective wafer. On the control layer wafer, the PDMS was spin-coated to achieve a layer thickness of approximately 30 µm. Both wafers were cured at 60 °C for 45 min. The flow layer was cut from the wafer, and the in- and outlets were punched using a 1-mm hole puncher (Ted Pella, Inc., USA). The flow layer was aligned on top of the control layer using an Olympus stereomicroscope. The layers were cured together at 60 °C overnight. The chip was then cut from the wafer. For interfacing with the FCB, the chip was plasma-bonded using a plasma cleaner (model CUTE, Femto Science, South Korea) to a glass slide 3 cm × 6 cm × 1 mm in size with 1-mm-diameter powder-blasted holes in the locations of the control channel inlets. For the unmounted chip used in the cell experiments, the inlets for the control channels were punched using a 0.75-mm hole puncher (Harris Uni-core) before plasma-bonding the chip to a 1-mm-thick glass microscope slide.

Approximately, 80% of all the mLSI MFBBs that were fabricated had at least 60 out of 64 (93–100%) fully independently operable chambers. The reason why in some cases not all 64 chambers were independently operable is that the valve membrane was not even in thickness over the entire chip. This resulted in areas where the pressure in the control channels was insufficient to close the valve fully, or if the pressure was increased, flow and control channel crossings that were supposed to remain open started to close off. Generally, chips that were cut from the center of the control layer wafer did not suffer from this issue, indicating that the main underlying cause is the photoresist being slightly thicker at the edges.

### Liquid dosing MFBB with a high dynamic range

Fabrication of the dosing MFBB consisted of modeling the 3D geometries in Autodesk Inventor and then generating computer-aided manufacturing files using Autodesk HSM. This was then sent to a Datron Neo computer numerical control (CNC) milling machine. The MFBB layers were made from 2-mm-thick PMMA stock. A total of three single-sided layers and one double-sided layer were machined, each measuring 30 mm by 30 mm by 2 mm. The hydraulic resistors on layer ①, as shown in Fig. 2d, varied in dimensions depending on hydraulic resistance. The high-resistance channels were 250 µm wide and 100 µm high, the medium-resistance channels were 250 µm wide and 180 µm high, and the low-resistance channels were 500 µm wide and 180 µm wide. The control lines were milled into layer ④ as shown in Fig. 2e, with channels that were 500 µm wide and 500 µm high. The valves, as shown in Fig. 2b, consisted of a bottom and top section (layers 2 and 3) with a 0.25-mm Viton rubber sheet (ACME rubber) clamped between the two using a 30% clamping ratio. The valve outer diameter was 3.5 mm, and each half was 200 µm high. The individual PMMA layers were solvent-bonded using a procedure adapted from the work of Ogilvie et al.^39^. Each side of the layers intended to form a bonded interface was exposed to chloroform (Sigma-Aldrich) vapor for 4 min, followed by being aligned and pressed together in a custom holder using a heated hydraulic press. The press was preheated to 65 °C, and the applied pressure was 100 N cm^−2^. After 20 min, the temperature was reduced to room temperature over 10 min by water cooling. The bonded chips were then left overnight before being used. Inlet and outlet tubing was connected using an NOA 81 optical adhesive.

### FCB and auxiliary parts

The FCB, MFBB clamps, and EIB were designed in SolidWorks^®^ (2018). The FCB consisted of five layers and a flexible membrane. The five layers were required for valve integration, channel bridging and channel sealing. The flexible membrane was required for valve actuation. The FCB channels used for controlling the MFBB were 300 µm wide by 400 µm high. The FCB channels controlling the FCB valves were 500 µm wide by 500 µm high. All channels were micromilled. The integrated valves were in a “normally closed” configuration and fit a footprint of 4 mm^2^. Areas below the MFBB microchambers were kept channel-free to prevent image distortion. The clamps were designed with asymmetrical bolt holes to fit together in a cog-like, space-saving manner (see Fig. S3). The clamps contained eight bolt holes in total, six of which were on the side where the O-rings need to be compressed to form a seal between the FCB and the MFBB. The EIB had 16 holes for tubing (13 for MFBB control and 3 for chip enabling) and two bolt holes (see Fig. S3).

FCB fabrication was outsourced to Micronit Microtechnologies (The Netherlands). The five layers were made from thermoplastic polystyrene and the flexible membrane from elastomeric SEBS. All channels in the layers were micromilled. Holes in the membrane for interlayer channel connections were created using a drag knife on a CNC machine. The layers were bonded together by thermal compression bonding. As a final step, the bolt holes were drilled, and the outside contour was milled.

In total, six FCBs were fabricated, of which three passed visual inspection. Of these three, the first one used for experimental testing was fully functional and therefore was used for all experiments presented in this article. The same FCB and auxiliary parts remained fully functional throughout the series of experiments for platform testing, characterization, and program optimization.

### Automation setup

All of the valves in the MFBBs and the FCB were driven by pneumatic actuation. Solenoid valves (Festo, The Netherlands), which were hooked up to a pressurized airline via a pressure regulator (Festo, The Netherlands), were used to switch between pressurized air (approximately 1.5 bar relative pressure) and atmospheric pressure (0 bar relative pressure). The solenoid valves were controlled through a custom LabView (2017, National Instruments, USA) program via an Easyport (Festo, The Netherlands) interface. The flow through the MFBBs was controlled using a pressure pump (Fluigent, Germany) and set using the aforementioned LabView program.

The custom LabView program contained functions for automated coating and filling of the channels and chambers in the mLSI MFBB. Furthermore, scripts to control different MFBBs on a single FCB (e.g., the mLSI and dosing MFBBs) could be loaded and run. In its current form, the platform still requires an experienced user for robust, leak-free assembly due to the variable O-ring compression described in the fabrication results section. However, once assembled, the operation of the platform is simple, and it can even be left unattended while a function or script is running. For experiments not presented in this article, four persons with no previous experience in microfluidics were able to successfully operate unmounted mLSI MFBBs similar to the one presented here after having had one introductory training session and a few independent tries on their own.

### Platform assembly

Nine 1-mm-diameter stainless steel pins (ERIKS BV, The Netherlands) were inserted into the nine corresponding holes in the FCB for MFBB alignment. Pieces of Tygon^®^ tubing (Metrohm, The Netherlands) with an outer diameter of 2.3 mm were inserted into the EIB on one end, filled with DI water, and hooked up to the solenoid valves on the other end. Next, the MFBBs were aligned on the FCB and then clamped into place. For an airtight seal, FKM O-rings with an inner diameter of 0.74 mm (ERIKS BV, The Netherlands) were used. The clamp was fastened using M2 hex bolts (DIN 934) inserted from the bottom of the FCB and tightened at the top with M2 nuts (RVS Paleis BV, The Netherlands). The pressure in the tubing for the MFBB control channels was increased to 1.6 bar by switching the solenoid valves that pushed the water through the FCB, filling the MFBB control channels. Water-filled control channels prevented air bubbles from forming at the valves in the mLSI MFBB flow layer during operation.

### Flow rate measurements

The flow rates for FCB valve characterization and the mLSI MFBB pressure retention experiment were measured using an L and an S flow sensor (Fluigent, Germany), respectively, and recorded with a custom LabView (2017, National Instruments, USA) program.

### Cell culture

In preparation for cell seeding, HUVECs (Lonza, Switzerland) or GFP-expressing HUVECs (Angio-Proteomie, USA) were cultured in collagen I-coated T75 flasks (CELLCOAT®, Greiner Bio-One) until reaching approximately 80% confluency. These cells were then trypsinized, centrifuged, and resuspended in endothelial growth medium (EGM) (Cell Applications, Inc., CA, USA) containing 25 mM hydroxyethyl piperazine-ethanesulfonic acid (HEPES). The cell suspension was filtered through a 40-µm pore-size filter (BD Falcon^™^) and then seeded in the previously prepared mLSI MFBB.

### mLSI MFBB preparation and cell seeding

Prior to cell seeding, the mLSI MFBB was prepared by selectively coating the flow channel walls to reduce cell adhesion and protein absorption and by coating the chamber walls to promote cell adhesion. The MFBB was exposed to oxygen plasma using a plasma cleaner (model CUTE, Femto Science, South Korea) to functionalize the surface with silanol groups. Next, all control channels and all flow channels were filled with sterile, filtered DI water. Keeping the chambers closed off, 100 µg/mL PLL-g-PEG (poly(l-lysine) poly(ethylene glycol)) (SuSoS, Switzerland) in PBS (Sigma-Aldrich) was flushed through all the channels and kept at room temperature for half an hour. Next, 0.1 mg/mL rat tail collagen I (Corning Life Sciences) in PBS was used to purge the PLL-g-PEG solution and then fill the chambers. The collagen solution has flowed through the chambers for 3 min, and the chip was then incubated for 1 h at 37 °C in the on-stage microscope incubator. Finally, all of the chambers and flow channels were filled with EGM (Cell Applications, Inc., CA, USA).

The cells were seeded at 6 × 10^6^ cells/mL through a pipette tip inserted in one of the inlets. The open top of the pipette tip was connected to a 3D-printed plug (3D printer by Formlabs, The Netherlands) with a hole for tubing, as described by Rho et al.^40^. Upon hooking up the tubing to a pressure pump (Fluigent, Germany), the air pressure inside the pipette tip was increased to 50 mbar. The chambers were filled with cells sequentially and then closed off. Next, the channels were flushed with a 1× trypsin (Invitrogen) solution several times to remove cells adhering to the channel walls. Finally, the trypsin solution was purged with EGM, and the LabView program was set to exchange the medium in the chambers every 3 h.

### Incubation system

**Fig. 8:**
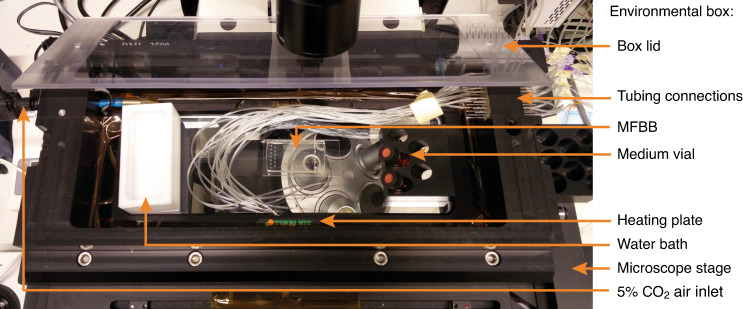
Top view of the environmental box mounted on an inverted microscope

A custom-built environmental box (Fig. 8) was used to maintain the MFBB under standard cell culture conditions (37 °C and 5% CO_2_). It was mounted on an MS-2500 motorized stage (Applied Scientific Instrumentation, USA), which was situated on a DMI 6000 m microscope (Leica Microsystems, Germany). The heating plate stage insert (Tokai Hit, Japan) was controlled using the aforementioned LabView program. The box had an internal volume of approximately 1.7 L. The removable lid provided easy access to the MFBB during tubing connection and cell seeding. Air with 5% CO_2_ was flowed into a humidity bath inside the box to keep the humidity inside high and thus prevent the MFBB from drying out. Additional wells filled with DI water were placed around the MFBB on the heating plate to further increase the humidity. The temperature was recorded using an NTC 10-kΩ thermistor (Vishay, USA) and documented using the same LabView program. A CO_2_ controller (Okolab, NA, Italy) supplied air with 5% CO_2_ to the environmental box. The advantages of this environmental box over a commercially available system are its low cost, easy chip access, and designated connections for microfluidic tubing. However, a disadvantage is that condensation occurs on the interior walls of the box, since these are cooler than the heating plate. While the environmental box is sufficient in its current state for demonstrating proof-of-principle cell culture, long-term cell culture would benefit from a uniform temperature and humidity distribution within the box. This can be achieved by encompassing the microscope in a temperature-controlled system instead of using a heating plate.

### Cell staining and imaging

HUVECs (Lonza, Switzerland) were fixed with 4% paraformaldehyde (Sigma-Aldrich) in PBS and subsequently permeabilized with 0.3% Triton-X (Sigma-Aldrich) in PBS. Next, 15 µL/mL of both ActinRed (Thermo Fisher Scientific) and NucBlue (Thermo Fisher Scientific) was added to the Triton-X solution to visualize the F-actin filaments and nuclei, respectively. Images were captured using a Leica DMI 6000 m microscope (Leica Microsystems, Germany) with a *p*E300^ultra^ LED illumination system (CoolLED, United Kingdom) and an RGB filter cube.

Live-cell images of the GFP-expressing HUVECs (Angio-Proteomie, USA) were taken with an EVOS FL cell imaging system using the GFP filter cube. The brightness and contrast of all images were adjusted using ImageJ.

## Supplementary information

Supplementary information

Supplementary video 1

Supplementary video 2

## References

[1] Wu MH, Huang SBin, Lee GBin (2010). Microfluidic cell culture systems for drug research. Lab Chip.

[2] Ye N, Qin J, Shi W, Liu X, Lin B (2007). Cell-based high content screening using an integrated microfluidic device. Lab Chip.

[3] Chen Q, Wu J, Zhang Y, Lin JM (2012). Qualitative and quantitative analysis of tumor cell metabolism via stable isotope labeling assisted microfluidic chip electrospray ionization mass spectrometry. Anal. Chem..

[4] Zhang Y, Zhang W, Qin L (2014). Mesenchymal-mode migration assay and antimetastatic drug screening with high-throughput microfluidic channel networks. Angew. Chem. Int. Ed..

[5] Woodruff K, Maerkl SJ (2016). A high-throughput microfluidic platform for mammalian cell transfection and culturing. Sci. Rep..

[6] Blazek M, Betz C, Nip Hall M, Zengerle R, Meier M (2013). Proximity ligation assay for high-content profiling of cell signaling pathways on a microfluidic chip. Mol. Cell. Proteom..

[7] Gómez-Sjöberg R, Leyrat AA, Pirone DM, Chen CS, Quake SR (2007). Versatile, fully automated, microfluidic cell culture system. Anal. Chem..

[8] Giobbe GG (2015). Functional differentiation of human pluripotent stem cells on a chip. Nat. Methods.

[9] Wu X (2016). In situ characterization of the mTORC1 during adipogenesis of human adult stem cells on chip. Proc. Natl Acad. Sci. USA.

[10] Giulitti S (2019). Direct generation of human naive induced pluripotent stem cells from somatic cells in microfluidics. Nat. Cell Biol..

[11] Brouzes E (2009). Droplet microfluidic technology for single-cell high-throughput screening. Proc. Natl Acad. Sci. USA.

[12] Wang BL (2014). Microfluidic high-throughput culturing of single cells for selection based on extracellular metabolite production or consumption. Nat. Biotechnol..

[13] Titmarsh DM (2017). Microfluidic screening reveals heparan sulfate enhances human mesenchymal stem cell growth by modulating fibroblast growth factor-2 transport. Stem Cells Transl. Med..

[14] Thorsen T, Maerkl SJ, Quake SR (2002). Microfluidic large-scale integration. Science.

[15] Gagliano O, Elvassore N, Luni C (2016). Microfluidic technology enhances the potential of human pluripotent stem cells. Biochem. Biophys. Res. Commun..

[16] Morton JA, Pietenpol WJ (1958). The technological impact of transistors. Proc. IRE.

[17] Unger MA, Chou HP, Thorsen T, Scherer A, Quake SR (2000). Monolithic microfabricated valves and pumps by multilayer soft lithography. Science.

[18] Zhang C (2019). Ultra-multiplexed analysis of single-cell dynamics reveals logic rules in differentiation. Sci. Adv..

[19] Dekker S (2018). Standardized and modular microfluidic platform for fast Lab on Chip system development. Sens. Actuators B Chem..

[20] Dekker S, Isgor PK, Feijten T, Segerink LI, Odijk M (2018). From chip-in-a-lab to lab-on-a-chip: a portable Coulter counter using a modular platform. Microsyst. Nanoeng..

[21] Ong LJY (2019). Self-aligning Tetris-Like (TILE) modular microfluidic platform for mimicking multi-organ interactions. Lab Chip.

[22] Rhee M, Burns MA (2008). Microfluidic assembly blocks. Lab Chip.

[23] Loskill P, Marcus SG, Mathur A, Reese WM, Healy KE (2015). μorgano: a Lego®-like plug & play system for modular multi-organ-chips. PLoS ONE.

[24] Vittayarukskul K, Lee AP (2017). A truly Lego®-like modular microfluidics platform. J. Micromech. Microeng.

[25] Shaikh KA (2005). A modular microfluidic architecture for integrated biochemical analysis. Proc. Natl Acad. Sci. USA.

[26] Heeren, H. Van, et al. Design guideline for microfluidic device and component interfaces. *Mfm*10.13140/RG.2.1.3318.9364 (2015).

[27] Hua Z (2006). A versatile microreactor platform featuring a chemical-resistant microvalve array for addressable multiplex syntheses and assays. J. Micromech. Microeng..

[28] Hansen CL, Sommer MOA, Quake SR (2004). Systematic investigation of protein phase behavior with a microfluidic formulator. Proc. Natl Acad. Sci. USA.

[29] Wang YBin (2017). An EWOD-based micro diluter with high flexibility on dilution ratio. Microsyst. Technol..

[30] Fan J, Li B, Xing S, Pan T (2015). Reconfigurable microfluidic dilution for high-throughput quantitative assays. Lab Chip.

[31] Ainla A, Gözen I, Orwar O, Jesorka A (2009). A microfluidic diluter based on pulse width flow modulation. Anal. Chem..

[32] Woodruff K, Maerkl SJ (2018). Microfluidic module for real-time generation of complex multimolecule temporal concentration profiles. Anal. Chem..

[33] Loessberg-Zahl, J. T. *Developing Microfluidic Tooling for 3D Cell-Culture*. PhD thesis (University of Twente, 2019).

[34] Grover WH, Ivester RHC, Jensen EC, Mathies RA (2006). Development and multiplexed control of latching pneumatic valves using microfluidic logical structures. Lab Chip.

[35] Toepke MW, Beebe DJ (2006). PDMS absorption of small molecules and consequences in microfluidic applications. Lab Chip.

[36] Van Der Meer AD, Van Den Berg A (2012). Organs-on-chips: breaking the in vitro impasse. Integr. Biol..

[37] Bhatia SN, Ingber DE (2014). Microfluidic organs-on-chips. Nat. Biotechnol..

[38] Huh D, Hamilton GA, Ingber DE (2011). From 3D cell culture to organs-on-chips. Trends Cell Biol..

[39] Ogilvie, I. R. G. et al. Reduction of surface roughness for optical quality microfluidic devices in PMMA and COC. *J. Micromech. Microeng.* **20**, 065016 (2010).

[40] Rho, H. S., Yang Y., Veltkamp H.-W., & Gardeniers, H. Direct delivery of reagents from a pipette tip to a PDMS microfluidic device. *Chips and Tips*. https://blogs.rsc.org/chipsandtips/2015/10/09/?doing_wp_cron=1604042654.1337950229644775390625 (2015).

