# Supplementary material for: Modular operation of microfluidic chips for highly parallelized cell culture and liquid dosing via a fluidic circuit board
Source: Microsyst Nanoeng. 2020 Nov 30;6:107. doi: 10.1038/s41378-020-00216-z (PMC8433198; doi:10.1038/s41378-020-00216-z)
Supplement: Supplementary file 1 — Supplementary information [file 41378_2020_216_MOESM1_ESM.docx]

**Supplementary Information**

**Modular Operation of Microfluidic Chips for Highly Parallelized Cell Culture and Liquid Dosing via a Fluidic Circuit Board**

**Authors:**
A.R. Vollertsen^1^, D. de Boer^2^, S. Dekker^1^, B.A.M. Wesselink^1^, R. Haverkate^1^, H.S. Rho^3^, R.J. Boom^4^,
M. Skolimowski^4^, M. Blom^4^, R. Passier^5^, A. van den Berg^1^, A.D. van der Meer^5^*, M. Odijk^1^*^†^

*Both authors contributed equally.

† Corresponding author. m.odijk@utwente.nl

**Authors’ affiliations:**
^1^ BIOS Lab on Chip Group, MESA+ Institute for Nanotechnology, University of Twente, Enschede, The Netherlands
^2^ Mesoscale Chemical Systems, MESA+ Institute for Nanotechnology, University of Twente, Enschede, The Netherlands
^3^ Institute for Technology-Inspired Regenerative Medicine, Maastricht University, Maastricht, The Netherlands
^4^ Micronit Microtechnologies, Enschede, The Netherlands
^5^ Applied Stem Cell Technologies, TechMed Centre, University of Twente, Enschede, The Netherlands

**1. mLSI MFBB control**

In this section the working principle of the mLSI MFBB control layer is described in detail. Fig. S1a shows the mLSI MFBB design with one control channel highlighted to show its path through the multiplexer. All control channels are dead-end channels so that they can be pressurized effectively and thus close the valves. Fig. S1b schematically shows the working principle of the combinatorial multiplexer. In this case, six flow channels can be independently addressed using four control channels. In the example the fourth channel is addressed by pressuring the top and bottom control channels. In the mLSI MFBB design, 64 flow channels are independently addressed using eight control channels. Fig. S1c schematically shows the filling of the control channels with deionized (DI) water. This step is necessary to prevent air in pressurized control channels from leaking into the flow channels. If the mLSI MFBB is mounted on the FCB, the DI water is supplied through the powder-blasted holes in the glass slide via the FCB. Alternatively, if the mLSI MFBB is used as a stand-alone device, the control lines are filled via hole-punched inlets in the PDMS (in this case there are no holes in the glass slide). Once pressurized, the water pushes out the air in the control channels through the PDMS. After 1-2 minutes, the controls channels are filled completely with water.


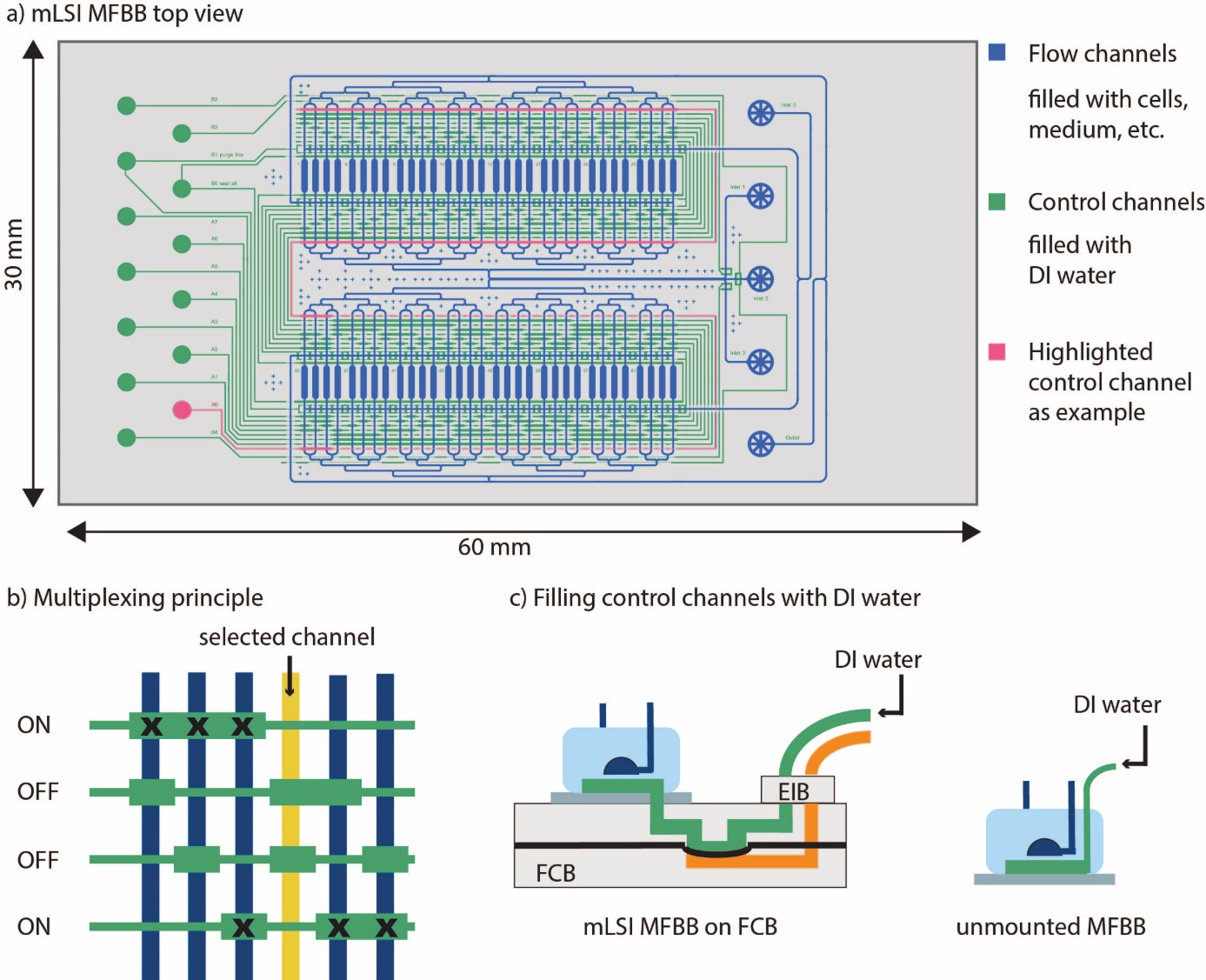


Fig. S1: Detailed mLSI MFBB control. a) Overview of the mLSI MFBB design with one of the control channels highlighted to show the channel path. b) Schematic multiplexing principle for six flow channels using four control lines. The ‘x’s represent closed valves. c) Schematic side view of the mLSI MFBB control channels being filled with DI water when the MFBB is mounted on the FCB (left) and when used as a stand-alone, unmounted chip (right).

**2. mLSI MFBB adjustments**

We made a few minor adjustments to our initial mLSI MFBB to accommodate cell culturing in the chambers. The initial mLSI MFBB (type I) and the adjusted MFBB (type II) are shown in Fig. S2a and 2d, respectively. Firstly, we re-routed the valve closing off the far left outlet (Fig. S2b) to the center inlet (Fig. S2e) to prevent cells from straying into the center inlet during seeding. The far left inlet was then only used as an outlet for a coating step during chip preparation. Secondly, we added pillars at the in- and outlets (Fig. S2b and Fig. S2e) to prevent the flow layer from sagging and curing to the control layer during fabrication. Thirdly, we made the chambers rectangular in shape to eliminate the crevices formed at chamber edges by reflowed photoresist. The difference in shape is shown in the two SEM (scanning electron microscope) images of the type I and type II wafer molds in Fig. S2c and Fig. S2f, respectively. Type I was used in the results showing the individual MFBB operation. Type II was used in the cell culturing results.


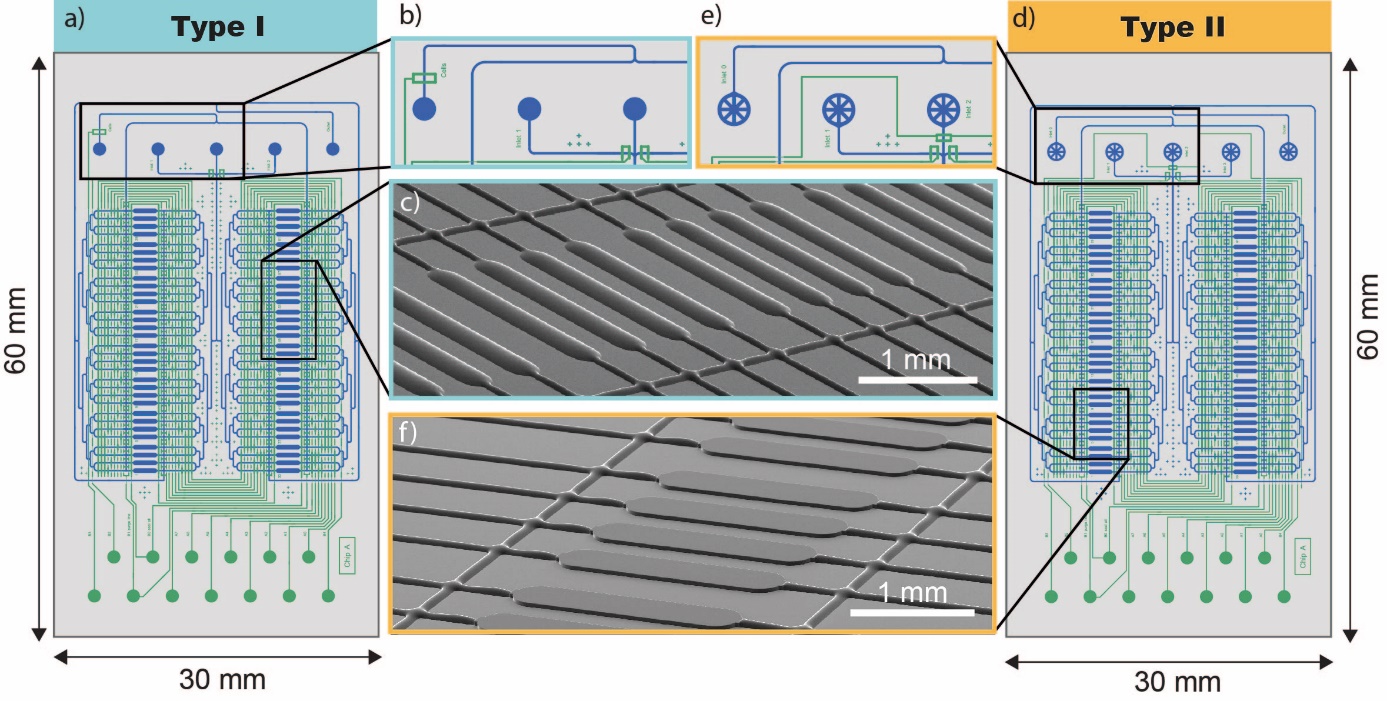


Fig. S2: Overview of the two mLSI MFBB versions. The flow channels are shown in blue and the control channels in green. a) Layout of the earlier version of the mLSI MFBB, referred to as type I. b) Close-up of the left outlet and two inlets. In this version, a valve closes off the outlet instead of the center inlet. c) Scanning electron microscope (SEM) image of the wafer mold showing a rounded profile of the chambers. d) Layout of the adapted mLSI MFBB, referred to as type II. e) Close-up of the left outlet and two inlets. Here, the valve closes off the center inlet instead of the left outlet. f) SEM image of the wafer mold showing a rectangular profile of the chambers and a rounded profile of the flow channels.

**3. Auxiliary components**

Fig. S3a shows the dimension of the external interconnection block (EIB) in a top and side view. The two larger through-holes at the top and bottom of the top view are designated for the bolts. The other 16 through-holes are designated for tubing which is hooked up to a pressure control system. Fig. S3b shows the top view of a clamp for a 3 cm × 6 cm MFBB. The through-holes along the perimeter are designated for the bolts. The asymmetric arrangement of the through-holes allows two adjacent clamps to be arranged in a cog-like fashion, thereby saving space. The clamp is designed to press down the outer edge of the glass slide of the mLSI MFBB against the FCB. This is shown in Fig. S3c in a side view. The glass slide (blue) is pressed against the O-rings (yellow) forming a seal. The 0.8 mm extension around the clamp through-holes act as a stopper and prevent too much force from being exerted on the glass itself. This in turn prevents the glass from cracking.


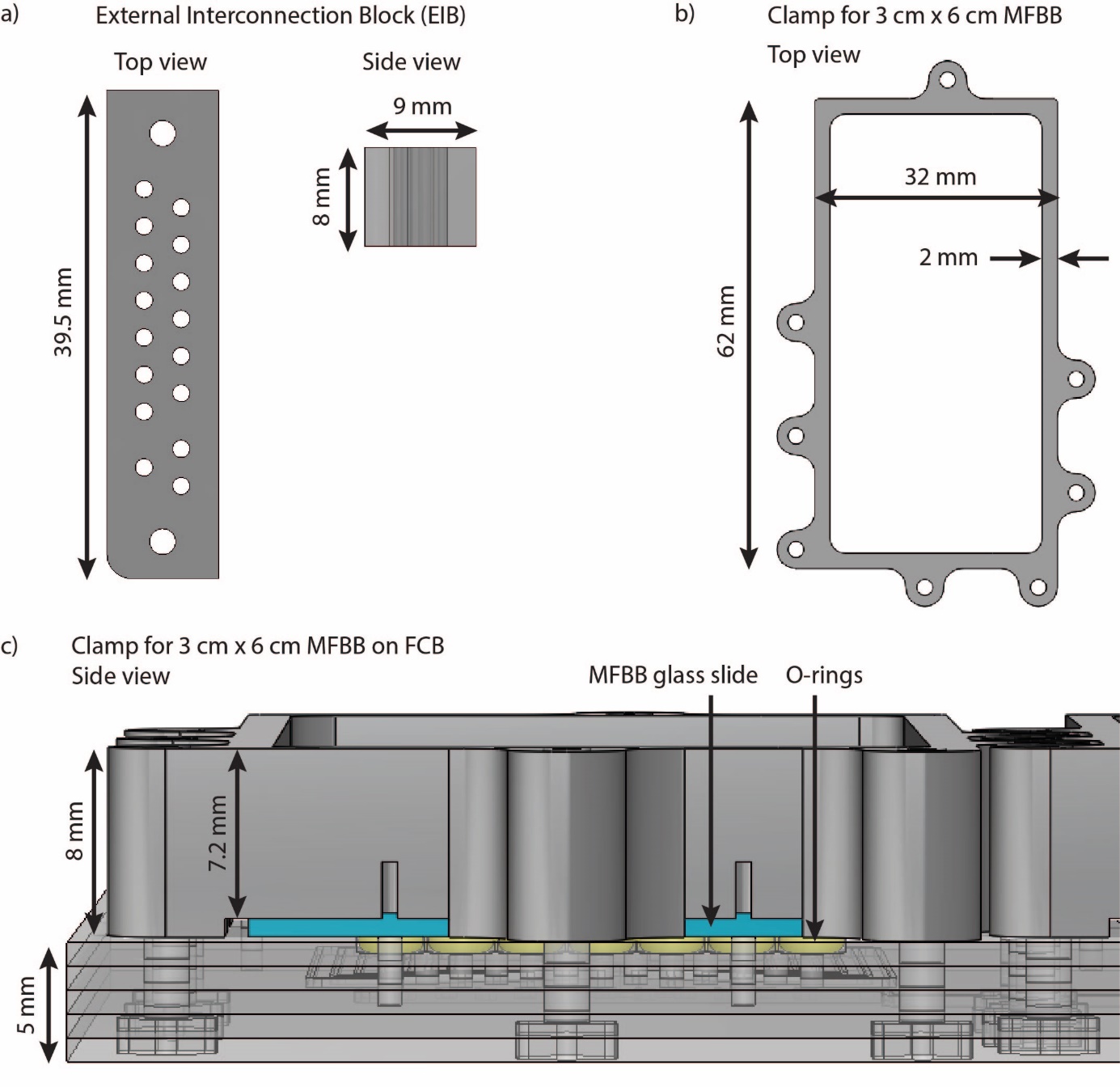


Fig. S3: Auxiliary parts for connecting the FCB with tubing and clamping the MFBBs. a) Dimensions of the EIB shown in a top and a side view. b) Dimensions of the mLSI MFBB clamp shown in a top view. c) Side view of the clamp pressing the MFBB glass slide against the O-rings in the FCB pockets.

**4. FCB valve characterization**

The FCB valves have a normally-closed configuration. Therefore, they are expected to be closed when the pressures on both sides of the membrane are equal. To confirm this, we characterized the closing behavior of a set of 13 FCB valves at 1400 mbar pump pressure and different gate pressures. All but one of the valves could be closed leak-free at gate pressures lower than or equal to the pump pressure. The reason for the residual leakage of the remaining valve is most likely a fabrication artefact in the form of non-uniform membrane tension.

The closing behavior of the FCB valves is characterized by measuring the flow of DI water through the FCB valves (Fig. S4a). Since the open valves have a very low hydraulic resistance, but the pump pressure at 1400 mbar is high, a hydraulic resistor is added between the DI water reservoir and the FCB. The hydraulic resistance of the resistor approximately 2 · 10^12^ Pa s m^-3^. The applied pump pressure is 1400 mbar, since this is typically used to close the valves in the mLSI MFBB. The flow rate is recorded while the gate pressure increases step-wise by 200 mbar, starting at -200 mbar and reaching 1600 mbar. Fig. S4b shows a bar graph of the flow rate for a set of 13 FCB valves at different gate pressures. Due to the low resistance of the open FCB valve, the flow rate at low gate pressures is mainly determined by the hydraulic resistor. As a consequence, the pressure on the top of valve membrane is effectively lower than the applied pump pressure. At 200 mbar gate pressure, the valve starts to pinch off the flow. At gate pressures at which the valve almost closes, the hydraulic resistance of the valve is much greater than that of the resistor. Therefore the pressure on the top of the valve membrane can be approximated with the applied pump pressure. Most of the valves (9 out of 13) are closed at 1000 mbar gate pressure.

To operate two MFBBs with different architectures via the FCB, it is crucial that the FCB can also save the open state of the mLSI MFBB valves (schematically shown in Fig. S4c). Fig. S4d shows two successive flow rate measurements through two chambers (1 and 4 in blue and purple, respectively) with an applied flow pressure of 50 mbar. Initially, the mLSI MFBB is enabled and the chamber is open. Subsequently, the MFBB is disabled and MFBB control lines which would close off the chamber are pressurized (indicated by the green line). At this point the time it takes for the chamber to close is measured. For chamber 1, the flow rate after 16 min is still at 95% of the initial flow rate. For chamber 4, the flow rate decreases to nearly 0 µL/min after 7 s, due to one of the FCB valves of the chip enable being slightly leaky (corresponds to valve 8 in Fig. S4b) at the applied pressures (p_FCB_ = p_MFBB_ = 1.7 bar). The leakage can be ameliorated by further increasing the FCB control channel pressure. Alternatively, the MFBB can be re-enabled every few seconds to reset the initial valve state.


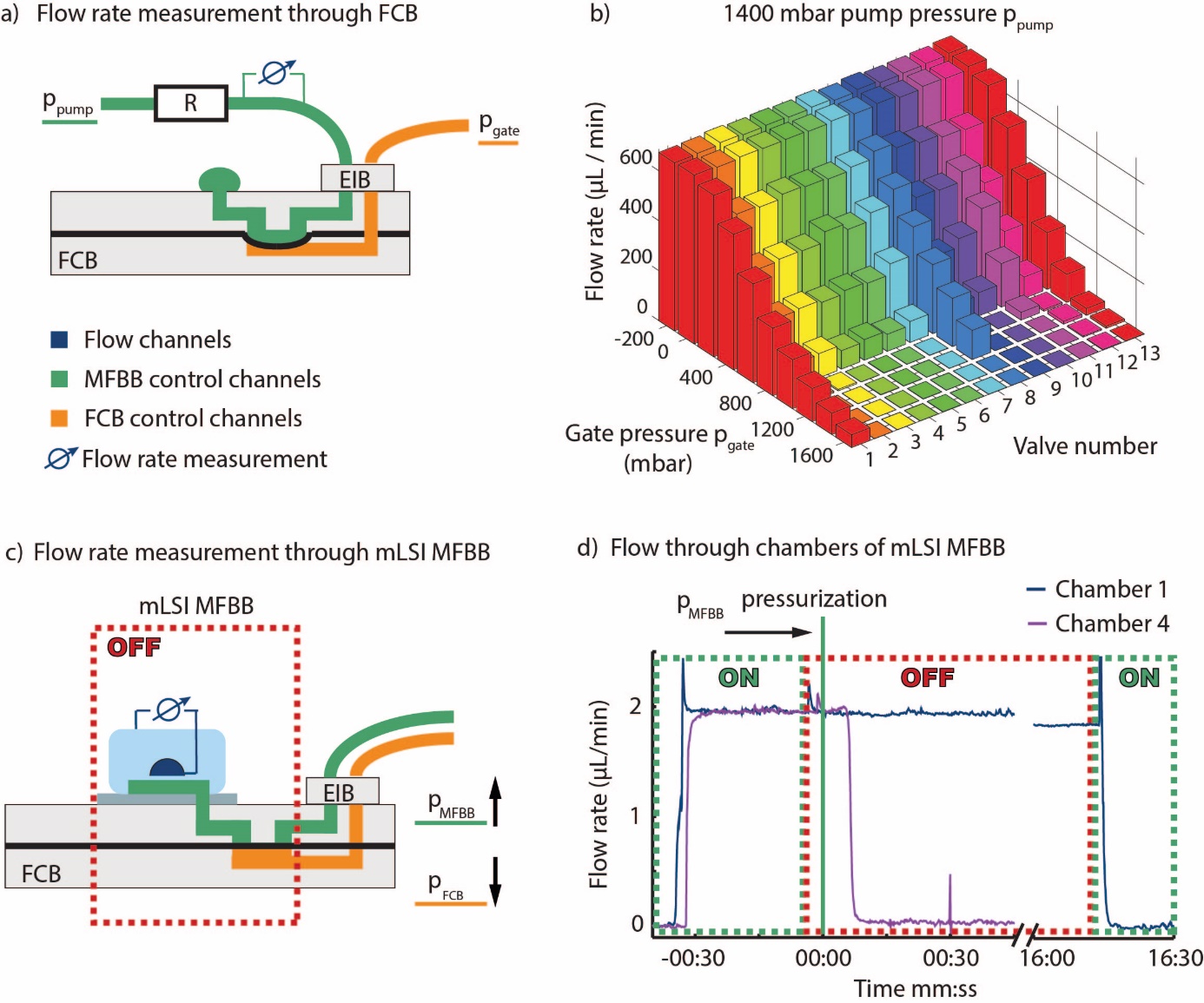


Fig. S4: a) Schematic view of the flow rate measurement. b) Bar graph showing the closing behavior of the FCB valves at 1400 mbar pump pressure and different gate pressures. c) Schematic showing the flow rate measurement through the mLSI MFBB with 64 chambers. d) Measured flow rate through chambers 1 (blue) and 4 (purple) while the mLSI is enabled (ON), subsequently disabled (OFF) and valves which would close the chambers are pressurized (green line) on the other side of the MFBB enabler.

**5. Dosing MFBB characterization**

The characterization of the dosing MFBB was performed using a FESTO easy port to actuate the control lines, a Fluigent EZ Flow to apply pressure to the liquid reservoirs, and a Fluigent model L flow rate platform meter to record both flow rate and metered volume. Water was used as the fluid, and pressure ranges from 200 mbar to 600 mbar were tested, as well as valve actuation times from 0.5 seconds to 10 seconds. At pressures lower than 270 mbar or actuation times shorter than 1 second instability in the metered flow can occur, and so these were used as the minimum values for subsequent experiments.

The first device characterization consisted of 10 repeats of opening and closing valves to direct water through one of the devices hydraulic resistor channels. An example of the flow rate data recorded by the Fluigent flowmeter can be seen in Fig. S5. The area under the flow rate time graph is the metered volume, and so varying the time that a valve is open for affects the volume of metered fluid. The metered volume at various pressures can be seen in Fig. S6 and 7 for both 1 second and 5 second opening times and based on the mean of 10 repeats. The hydraulic resistances of the metering resistors were calculated using Equation 3 based on the mean flow rate while the valves were open. The resulting resistances from a 5 second opening and closing time with an applied pressure of 600 mbar can be seen in Tab. S1.

To determine the dynamic range of the MFBB, the metered volume for steps from 1 to 10 second valve opening times was measured 3 times for each resistor at 300 mbar. The dynamic range of the system can be seen in Figure 6a, which shows the mean metered volume for different channels for durations of 1 to 10 seconds. The plot shows generally linear behavior, which allows for a predictable volume to be metered through a combination of pulses from the high, medium, and low resistance channels. There is variability in the metered volume which is likely responsible for the lower R^2^ values for the high resistance channels compared to the low resistance channels, however this is challenging to accurately characterize given the accuracy of the flow rate meter used to record these values. A larger factor which is problematic for repeated use of the system is the inconsistency in hydraulic resistance of channels. This is evident by the difference in the metered volumes of two low resistance channels. Low resistance channel 2 metered over twice the volume of low resistance channel 1 in Fig. S7, despite the only 1.52 time difference in hydraulic resistance as seen in Tab. S1. This is primarily caused by bubbles trapped in the valves, the location of which remains mostly consistent during usage of the MFBB, until the device is flushed with air and then refilled. An example of this can be seen in Fig. S8, and should be addressed in subsequent designs by removing the cavities in which bubbles can get stuck.


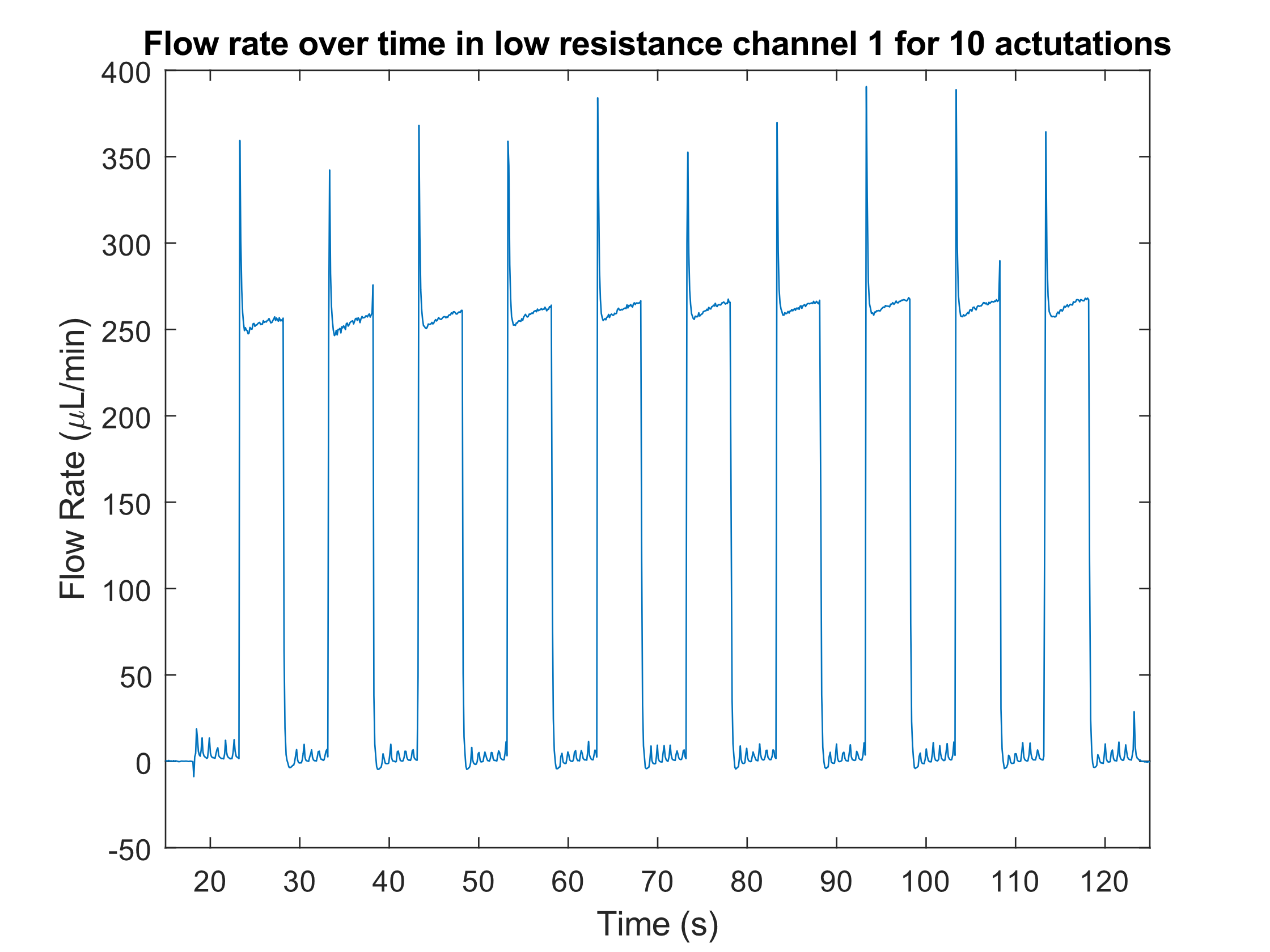


Fig. S5: An example of flow rate data for 10 repeats of a 5 second valve actuation through low resistance channel 1 in the dosing MFBB.

Tab. S1: Hydraulic resistances in Pa s m^-3^ for the resistors in the dosing MFBB.

| Low resistance 1 | Low resistance 2 | Medium resistance 1 | Medium resistance 2 | High resistance 1 | High resistance 2 |
| --- | --- | --- | --- | --- | --- |
| 4.04 · 10^9^ | 2.65 · 10^9^ | 4.95 · 10^9^ | 4.14 · 10^9^ | 2.67 · 10^10^ | 3.02 · 10^10^ |


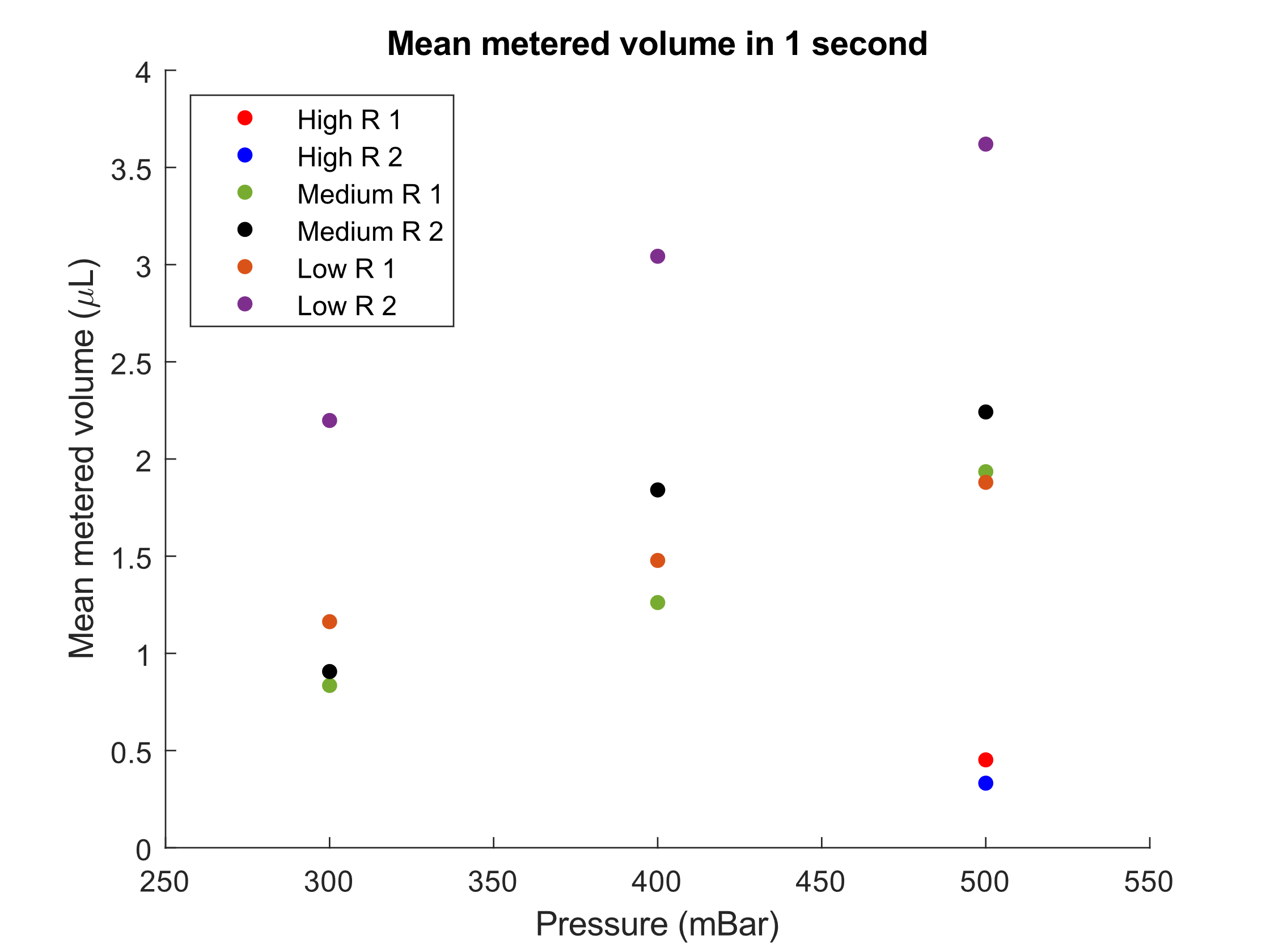


Fig. S6: Mean metered volume for 10 repeats of a 1 second valve opening time at various pressures. Data from high resistance channels 1 and 2 is not present at lower pressures due to the range of the flow meter which was used.


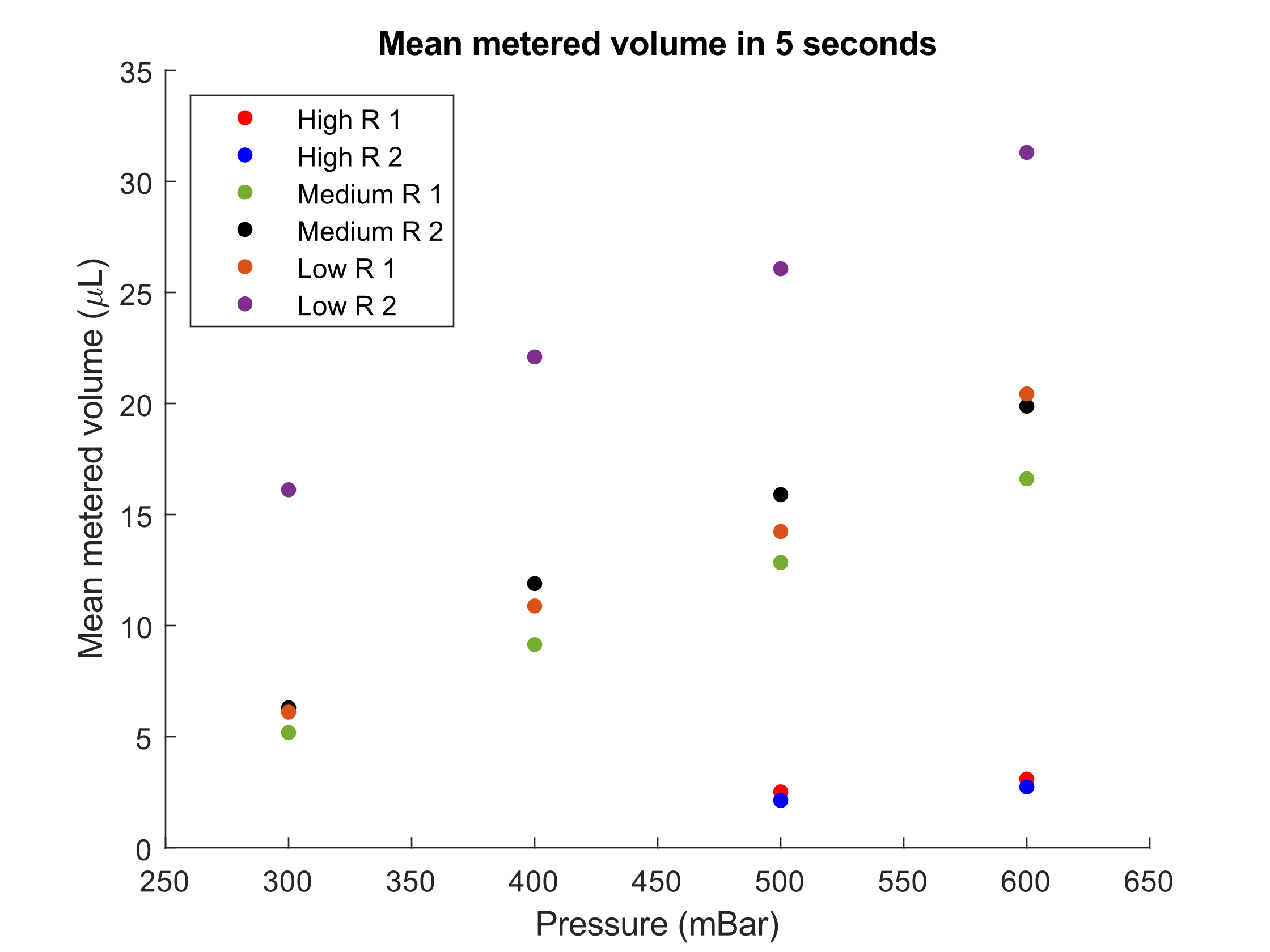


Fig. S7: Mean metered volume for 10 repeats of a 5 second valve opening time at various pressures. Data from high resistance channels 1 and 2 is not present at lower pressures due to the range of the flow meter which was used.


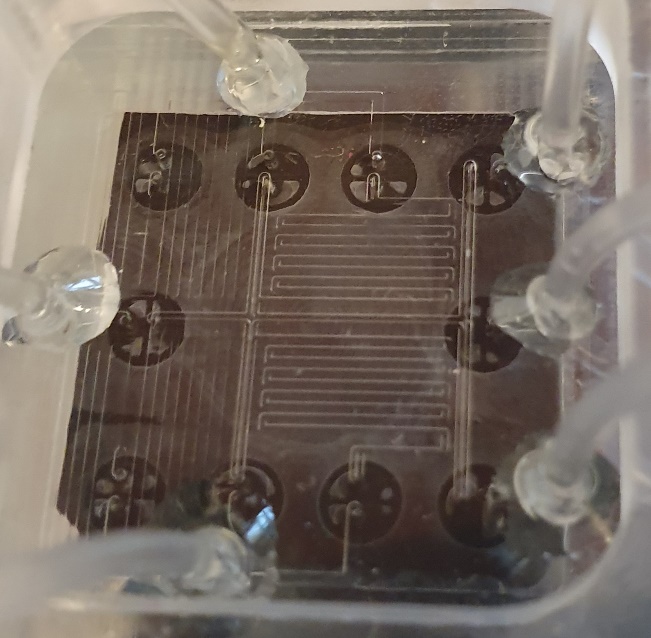


*Fig. S8: A photograph showing bubbles trapped in the valves.*
